# Nanoscratch-Informed Micro-to-Macro Surrogate Modeling of Fracture Properties in Nano-Silica Modified Portland Cement Paste

**DOI:** 10.3390/nano16211440

**Published:** 2026-11-06

**Authors:** Xiangong Zhou, Manhui Liu, Xiancheng Zhou, Weikang Kong

**Affiliations:** 1 School of Urban Construction, Hangzhou Polytechnic, Hangzhou 311402, China; zhouxiangong@hzpt.edu.cn; 2 Zhejiang Scientific Research Institute of Transport, Hangzhou 311402, China; 3 College of Architectural Engineering, Shanxi Vocational University of Engineering Science and Technology, Jinzhong 030619, China; 4 Intelligent Transportation System Research Center, Southeast University, Nanjing 210096, China; 230208814@seu.edu.cn

**Keywords:** cement paste, nano-silica, nanoscratch, surrogate modeling, XGBoost-SHAP

## Abstract

Macroscopic fracture testing of nano-modified cement paste requires the labor-intensive fabrication of notched beams. This study establishes a micro-to-macro surrogate modeling framework mapping localized nanomechanical scratch signatures to macroscopic fracture resistance. A database was compiled for pure Portland cement paste (*w*/*c* = 0.30, 28-day curing) across seven nano-silica (NS) dosages (0.0–3.0%). Across 21 independent physical beam specimens (*N_macro_* = 21) and 470 validated phase-segmented nanoscratch observations (*N_micro_* = 470), localized descriptors were acquired across unhydrated clinker, hydration products, and the clinker–hydrate interfacial transition zone (micro-ITZ). Within a leak-free pipeline, an XGBoost surrogate model was trained using specimen-grouped nested cross-validation (Nested GroupKFold), achieving cross-validated *R*^2^ values of 0.878 ± 0.024 (95% CI [0.834, 0.919]) for macro-fracture toughness ($K_{\mathrm{IC}}^{\mathrm{macro}}$) and 0.865 ± 0.028 (95% CI [0.815, 0.912]) for fracture energy (*G_f_*). Leave-one-dosage-out validation demonstrated that when provided with the target mix’s in situ nanoscratch fingerprint, the model successfully tracked the non-monotonic fracture evolution, including the peak at 2.0% NS and the agglomeration degradation at 3.0% NS. Bootstrap Tree-SHAP attribution identified interfacial micro-toughness ($K_{\mathrm{IC},\mathrm{ITZ}}^{\mathrm{micro}}$) as the primary predictive contributor (42.5% [95% CI: 37.8%, 47.1%]), revealing a model-suggested transition region around 0.80 MPa ⋅ m^1/2^ above which interfacial crack resistance associates with substantial macroscopic toughening. This framework provides an efficient surrogate approach for evaluating macro-fracture properties from micromechanical indicators.

## 1. Introduction

As one of the most widely used engineering materials in modern infrastructure construction, the service performance of cementitious materials directly affects structural safety and durability. However, hardened cementitious materials possess a typical multiscale heterogeneous structure, consisting of unhydrated cement particles, hydration products, the interfacial transition zone (ITZ), and multiscale pores. These microstructural phases exhibit distinct differences in chemical composition, structural morphology, and mechanical response, and they influence deformation, damage, and fracture processes through complex interactions. Therefore, establishing a multiscale correlation between microstructural features and macroscopic mechanical properties is a critical scientific issue for designing high-performance cementitious materials.

In recent years, nanomaterials have been widely applied to optimize the performance of cementitious materials due to their unique size effect, high specific surface area, and interface control capability. Among them, nano-silica (NS) is considered one of the most promising nano-reinforcement materials due to its excellent pozzolanic activity, nano-filling capacity, and hydration promotion effect [1,2]. The performance improvement of cementitious materials by nano-silica primarily originates from the filling effect, nucleation effect, and pozzolanic reaction [3]. Microscopic pores within the cement paste can be filled by nanoparticles, thereby reducing pore connectivity and enhancing material density. Meanwhile, abundant active nucleation sites are provided on the nanoparticle surface, accelerating the deposition of hydration products and promoting C-S-H gel formation. Furthermore, additional C-S-H gel is generated through the secondary pozzolanic reaction between active NS and calcium hydroxide (CH), which improves the internal structural stability of the material [4,5,6].

Recent studies have demonstrated that the mechanical properties and durability of cementitious materials can be effectively improved by nano-silica. Wang et al. [2] found that the pore structure of cementitious materials was significantly optimized and impermeability was enhanced by colloidal nano-silica. Further studies pointed out that the NS content exerts a significant influence on the cement hydration process and microstructural evolution; an appropriate content promotes hydration and improves performance, whereas an excessive content may reduce the reinforcement effect due to particle agglomeration [4,7]. However, existing research primarily focuses on macroscopic indicators such as compressive strength and durability, while an in-depth understanding of how nano-silica affects fracture behavior and the role of different micro-regions during damage processes remains lacking [8].

Cementitious composites are typical quasi-brittle materials whose fracture behavior involves microcrack initiation, steady tortuous propagation, and catastrophic failure. Compared with standard strength metrics, macroscopic fracture toughness ($K_{\mathrm{IC}}^{\mathrm{macro}}$) and fracture energy (*G_f_*) quantify the fundamental capacities to resist crack growth and dissipate fracture energy, serving as primary indicators for assessing crack propagation resistance [9]. Microstructural heterogeneity dictates macroscopic fracture paths: while the porous clinker–hydrate boundary acts as the preferential initiation locus [10], the intrinsic shear-cutting resistance and cohesive energy of the surrounding C-S-H gel govern post-peak crack tortuosity and bridge dissipation [11,12,13,14].

Establishing a quantitative predictive link between localized micromechanical properties and macroscopic fracture resistance represents a fundamental challenge. Traditional analytical micromechanical homogenization methods (such as Mori–Tanaka schemes, self-consistent models, and continuum damage mechanics) typically rely on idealized spherical inclusions and linear-elastic interfacial bonding assumptions [9,11]. Consequently, they struggle to capture the localized tangential plowing, plastic yielding, and non-monotonic agglomeration thresholds characteristic of multiphase nano-modified pastes. To overcome this limitation, obtaining in situ, multiphase mechanical signatures directly from individual constituent phases is essential [15].

In recent years, instrumented nanoscratch testing has emerged as a robust technique for probing localized damage and fracture evolution across heterogeneous micro-phases in cementitious and composite materials [16,17,18]. Unlike static nanoindentation, which records only normal compliance, continuous nanoscratch testing imposes combined normal and tangential loading. This enables the direct acquisition of multidimensional micromechanical indicators, including scratch hardness (*H_s_*), apparent friction coefficient (*μ*), elastic recovery rate (*E_r_*), scratch energy dissipation (*W_s_*), and local micro-fracture toughness ($K_{\mathrm{IC}}^{\mathrm{micro}}$), determined via scratch fracture mechanics [19,20].

In our previous investigations [16,17,18,19], nanoscratch testing was applied as a diagnostic micro-characterization tool to analyze local phase degradation under frost attack or to compare a single 2.0% NS mixture against the control paste. However, those prior studies were strictly observational and qualitative: nanoscratch data were utilized merely for post-mortem description rather than as predictive input features, and high-dimensional mapping connecting multiphase scratch signatures to macroscopic fracture resistance across continuous mix formulation gradients was never established [21].

Concurrently, data-driven machine learning (ML) paradigms have demonstrated exceptional capacity in capturing complex, non-linear property mappings across materials science [22,23], with general deep learning frameworks synthesized by Agrawal and Choudhary [24]. In the field of concrete and cementitious composites, machine learning algorithms have been increasingly applied to predict mechanical properties [25], durability, and multiscale behavior, as comprehensively reviewed by Gamil [26] and Hu et al. [27]. Moreover, recent studies have demonstrated that microstructural information can be integrated into data-driven models to evaluate critical mechanical properties in cementitious composites [25,28,29].

However, the vast majority of existing ML models still rely exclusively on macroscopic mix design proportions (e.g., water-to-cement ratio, mineral admixture percentages, and curing age) as input features [24,28]. When deployed across unobserved formulation regimes, these purely composition-based “black-box” models suffer from severe extrapolation failures; lacking in situ physical knowledge of microstructural development, they cannot anticipate non-monotonic performance reversals caused by physical limits such as nanoparticle agglomeration [7,29]. Although integrating microstructural descriptors into machine learning has been recognized as a viable strategy to enhance physical consistency [25,30], an interpretable, data-driven surrogate framework informed by multiphase nanoscratch signatures has not yet been established for fracture mechanics prediction in cement paste.

Therefore, combining multidimensional nanomechanical data obtained from nanoscratch testing with data-driven models holds promise for establishing multiscale predictive relationships between nanoscratch responses and macro-fracture toughness, thereby further revealing key nanoscale factors controlling fracture performance.

Based on this, a multiphase nanoscratch database for nano-silica reinforced cementitious materials was constructed in this study, incorporating the nanomechanical responses of unhydrated cement particles, hydration products, and ITZ regions. Data-driven models were developed to predict the macroscopic fracture energy (*G_f_*) and fracture toughness ($K_{\mathrm{IC}}^{\mathrm{macro}}$) of the materials. By comparing the predictive performance of different models and employing feature attribution methods to uncover key governing parameters, this study aims to establish a multiscale prediction framework ranging from nanoscale mechanical responses to macroscopic fracture properties, providing new theoretical and methodological support for the intelligent design of nano-reinforced cementitious materials.

## 2. Materials and Methods

### 2.1. Materials and Specimen Fabrication

Ordinary Portland cement (OPC) was utilized as the primary binder, and its chemical oxide compositions and basic physical properties are summarized in Table 1. The nano-reinforcement additive was a commercial aqueous colloidal nano-silica (NS) suspension (solid content 30.0 wt.%). The colloidal NS particles possessed an average primary spherical diameter of 20 ± 5 nm, a specific surface area of 160 ± 20 m^2^/g, a density of 1.20 g/cm^3^, and a suspension pH of 7.5.

A pure cement paste system was used to investigate the intrinsic microstructural evolution and phase transformations under nano-silica modification. The net water-to-binder (*w*/*b*) ratio was maintained constant at 0.30 across all formulations. Nano-silica was incorporated as an internal mass replacement for cement at dosages of 0.0%, 0.5%, 1.0%, 1.5%, 2.0%, 2.5%, and 3.0% by weight of the dry cement. The free water introduced by the aqueous colloidal NS suspension was precisely deducted from the batch mixing water to ensure that the effective *w*/*b* ratio remained strictly at 0.3. The detailed mixture proportions for all seven groups are listed in Table 2.

To achieve physical deagglomeration of the nanoparticles without chemical surfactants, the aqueous colloidal NS was dispersed directly into the batch water using an ultrasonic probe homogenizer (300 W, 40 kHz) for 15 min with pulse intervals (3 s active/2 s rest). An ice-water bath was applied during sonication to prevent temperature rise and pre-hydration. The cement powder was charged into an epicyclic paste mixer, followed by the addition of the sonicated NS-water suspension. Mixing was performed at low speed (140 rpm) for 120 s, followed by a 15 s pause to scrape down paste adhering to the bowl walls, and concluded with high speed (285 rpm) for 120 s.

Fresh paste was poured into standard prismatic steel molds (40 mm × 40 mm × 160 mm) and consolidated on a vibrating table for 30 s to eliminate entrapped air voids. The specimens were covered with wet plastic membranes to prevent moisture evaporation and maintained at 20 ± 2 °C for 24 h. After demolding, the prismatic specimens were cured in a standard curing chamber at 20 ± 1 °C and ≥95% relative humidity for 28 days before fracture and nanoscratch testing.

In this pure cement paste system, the interfacial transition zone (designated as micro-ITZ) is unambiguously defined as the intrinsic clinker–hydrate boundary transition zone between the unhydrated cement clinker core (UCP) and the surrounding outer hydration product (C-S-H gel) matrix.

### 2.2. Data Acquisition and Construction of Nanoscratch Database

To establish absolute experimental traceability and eliminate statistical pseudoreplication, the database was built by harmonizing standard baseline batches from the authors’ previous testing protocols [16,31] with a systematic experimental campaign conducted in this study. Crucially, in our prior studies [16,31], standard non-frost baseline tests were restricted to an identical water-to-cement ratio (*w*/*c* = 0.30) and evaluated only a single nano-silica dosage of 2.0% alongside the 0.0% control. Strict harmonization was enforced across all campaigns: (1) all specimens consisted of pure Portland cement paste (*w*/*c* = 0.30, no chemical admixtures); (2) all samples were cured under standard conditions (20 ± 1 °C, RH ≥ 95%) for 28 days without environmental damage; (3) all nanoscratch tests were executed on the same Nano Indenter G200, KLA Corporation, Oak Ridge, TN, USA, platform using identical Berkovich tip geometry, constant normal load (*F_n_* = 10 mN), velocity (0.5 μm/s), and track length (100 μm); and (4) macroscopic fracture parameters were obtained using identical beam geometries and RILEM testing procedures.

A statistical sample (one row in the database) is explicitly defined as a standardized, phase-resolved microstructural observation vector. Each row pairs the localized nanomechanical descriptors extracted from a specific nanoscratch segment with the macroscopic fracture parameters ($K_{\mathrm{IC}}^{\mathrm{macro}}$ and *G_f_*) measured on the exact same physical pre-notched beam specimen. As detailed in Table 3, baseline standard-cured records from previous non-frost control batches (Series A: 0.0% and 2.0% NS, comprising 6 beam specimens and 132 validated micro rows) were harmonized with the newly executed campaign (Series B: 0.5%, 1.0%, 1.5%, 2.5%, and 3.0% NS, comprising 15 beam specimens and 338 validated micro rows, accounting for 71.9% of the database). Across all seven dosages, exactly 3 replicate beam specimens were tested per group (21 macroscopic specimens in total). For each beam, 1 polished section was prepared adjacent to the fracture ligament, on which 4 target UCPs were selected. Two nanoscratch tracks were executed across each UCP–ITZ–matrix region (8 tracks per specimen, 24 tracks per dosage, and 168 tracks overall). Each track generated 3 phase-resolved segments, yielding 504 raw observations. Following physical validity checks and multivariate outlier screening (average exclusion rate of 6.7%), exactly 470 validated rows were finalized.

The local mechanical responses acquired via nanoscratch testing can effectively reflect the resistance to deformation, damage accumulation, and energy dissipation of different micro-phases. Specifically, the local stiffness and load-bearing capacity of original clinker particles are reflected by the unhydrated particle regions; network density and local deformation behavior of C-S-H gels are described by the hydration product regions; and bonding states at the clinker–hydrate micro-interface (micro-ITZ) along with damage resistance in potential crack initiation zones are directly characterized by the ITZ parameters.

The input variables of the database cover the scratch hardness (*H_s_*), apparent friction coefficient (*μ*), maximum/residual scratch depth (*h*_max_, *h_r_*), elastic recovery rate (*E_r_*), scratch energy dissipation (*W_s_*), and micro-fracture toughness ($K_{\mathrm{IC}}^{\mathrm{micro}}$) derived from scratch fracture mechanics inversion across the three phase regions. The elastic recovery rate (*E_r_*) and scratch energy dissipation (*W_s_*) are calculated as follows:

(1) $$E_{r}=\frac{h_{\max}-h_{r}}{h_{\max}}\times 100\%$$

(2) $$W_{s}=\int_{0}^{L}F_{t}\left(x\right)\;\mathrm{dx}$$

where *F_t_*(*x*) is the instantaneous tangential force, and *L* is the scratch length. The calculation method for micro-fracture toughness ($K_{\mathrm{IC}}^{\mathrm{micro}}$) is detailed in Section 2.2.

The output variables of the database were selected as macroscopic fracture parameters reflecting the crack resistance of the material, including fracture energy (*G_f_*) and macro-fracture toughness ($K_{\mathrm{IC}}^{\mathrm{macro}}$). Compared with a single overall indicator, the local phase evolution process under the influence of NS can be captured more comprehensively by the multiphase nanomechanical feature matrix, which lays a solid data foundation for subsequent data-driven models to establish high-dimensional non-linear mapping relationships.

### 2.3. Micro- and Macro-Fracture Property Testing

#### 2.3.1. Micro Fracture Property Testing

To accurately capture the in situ nanomechanical response and local fracture resistance of unhydrated cement particles (UCPs), hydration products (dominated by C-S-H gel), and the interfacial transition zone (ITZ) within cementitious materials, high-precision nanoscratch testing technology was employed. The nanoscratch testing methodology, specimen preparation, and data processing procedures used in this study were kept consistent with the authors’ previous research, with relevant details reported in earlier literature [15,27].

Considering the high sensitivity of the test to surface flatness, specimens were embedded in epoxy resin via vacuum impregnation, sequentially ground with silicon carbide sandpapers ranging from #240 to #4000 under water lubrication, and finely polished using diamond pastes with particle sizes of 3 μm, 1 μm, and 0.25 μm combined with anhydrous ethanol until a root-mean-square surface roughness of *R_q_* < 20 nm was achieved over a 50 μm × 50 μm area.

Testing was performed on a high-precision nanomechanical testing platform (Nano Indenter G200) equipped with a Berkovich diamond indenter (nominal tip radius of curvature *R* ≈ 100 nm). All nanoscratch experiments across all specimens were conducted exclusively under continuous loading mode (progressive linear ramp loading). Over the scratch length of *L* = 100 μm at a constant scratching velocity of *v* = 0.5 μm/s (total scratching duration of 200 s), the applied normal load was linearly increased from an initial contact preload of *F_n_*_,min_ = 0.2 mN to a peak normal load of *F_n_*_,max_ = 10 mN at a constant loading rate of *dF_n_*/*dt* = 0.05 mN/s. A high data acquisition frequency of 200 Hz was maintained, capturing 400 discrete data points per micrometer along the track. Prior to each scratch run, thermal drift was monitored until it stabilized below 0.05 nm/s. The tip area function *A_c_*(*d*) and perimeter function *p*(*d*) were calibrated on a standard fused silica reference block (*E* = 72.0 GPa, *H* = 9.2 GPa) following the Oliver–Pharr method. Tip wear and system compliance were verified every 24 scratch tracks; deviations in contact area exceeding ±4% triggered tip ultrasonic cleaning and recalibration.

**Figure 1:**
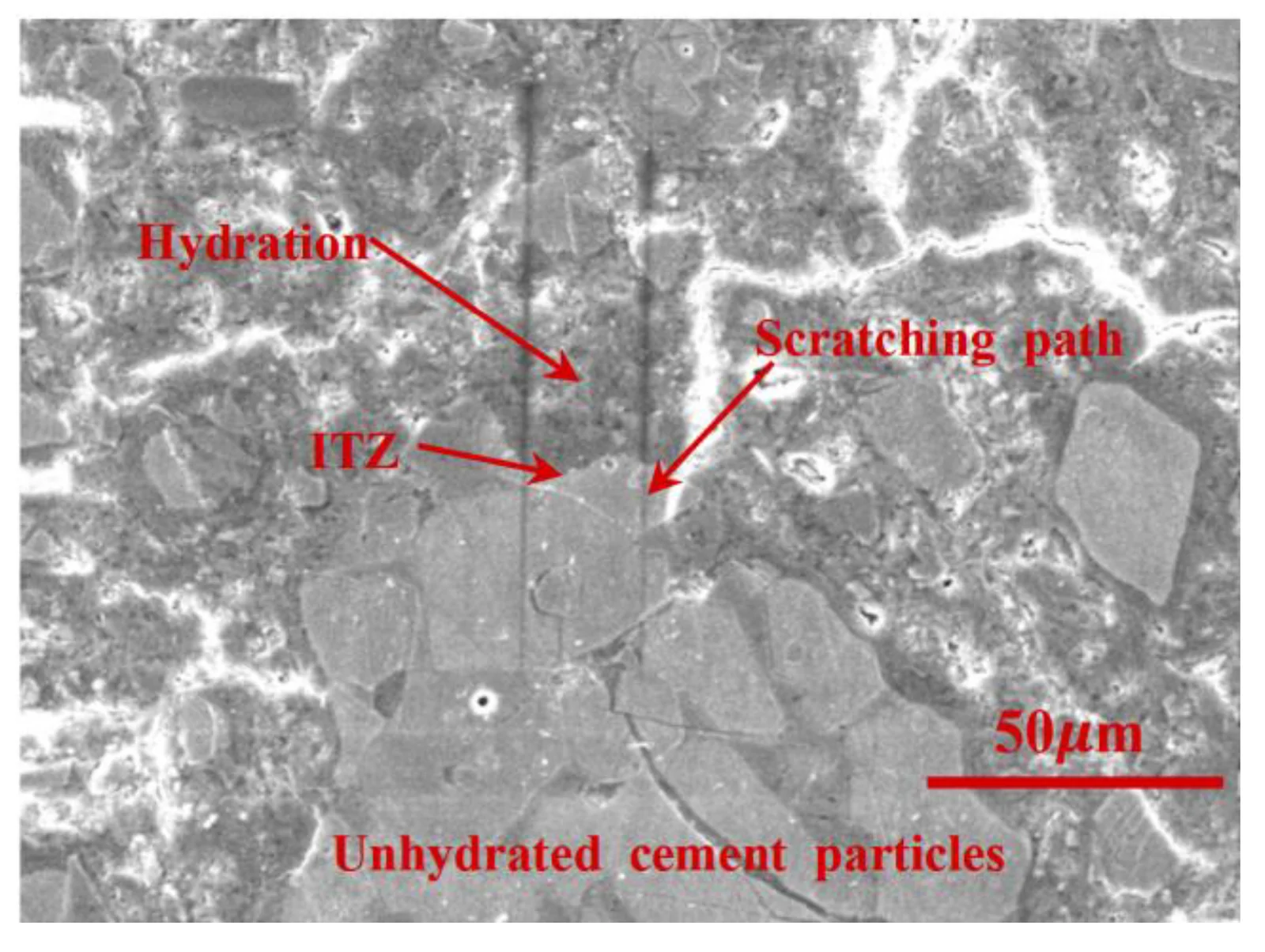
Scratching path on unhydrated cement particles, ITZ, and hydration.

The scratching path traversing unhydrated cement particles, the micro-ITZ, and the hydration product matrix is illustrated in Figure 1. Phase segmentation along each 100 μm progressive scratch track was determined using an objective multi-criteria protocol combining optical microscopy, AFM scanning, and post-scratch backscattered electron (BSE) imaging: (1) unhydrated cement particles (UCPs) were confirmed by bright backscattered clinker phase contrast, characterized by scratch penetration depths *d* < 120 nm and scratch hardness *H_s_* > 4.5 GPa; (2) hydration product matrix (predominantly C-S-H gel) was identified by intermediate gray matrix contrast, with *d* ranging from 180 nm to 320 nm and *H_s_* between 1.1 GPa and 2.2 GPa; (3) the clinker–hydrate interfacial transition zone (micro-ITZ) was identified within a 15–25 μm annular boundary surrounding the UCP core, featuring elevated penetration depths (*d* ≈ 300–450 nm) and reduced hardness (*H_s_* < 1.4 GPa). To eliminate boundary artifact effects, a buffer exclusion margin of 3.0 μm was trimmed from both sides of every phase interface. Considering a maximum scratch depth of *d* ≈ 450 nm, the lateral fracture process zone radius was estimated as *R_p_* ≈ 3*d* ≈ 1.35 μm, ensuring that the probed stress volume remained strictly contained within each segmented micro-phase.

The local micro-fracture toughness ($K_{\mathrm{IC}}^{\mathrm{micro}}$) was derived using a scratch energy balance model. During continuous scratching, the tangential work performed by probe advancement was converted into plastic deformation energy and fracture energy dissipated during new surface formation. Based on scratch fracture mechanics theory, the local micro-fracture toughness $K_{\mathrm{IC}}^{\mathrm{micro}}$ (MPa ⋅ m^1/2^) can be expressed as a function of the tangential force and probe contact geometry:

(3) $$K_{\mathrm{IC}}^{\mathrm{micro}}=\frac{F_{t}}{\sqrt{2p\left(d\right)A_{c}\left(d\right)}}\times 10^{-3/2}$$

where *F_t_* is the representative steady tangential force resolved within the segmented phase window (N), *p*(*d*) is the contact perimeter (mm), *A_c_*(*d*) is the horizontally projected load-bearing contact area (mm^2^), and *d* is the penetration depth. For the self-similar Berkovich indenter utilized under the present scratching orientation, the product of contact perimeter and horizontally projected area obeys the ideal geometric scaling relation *p*(*d*)*A_c_*(*d*) = 2.3432 *d*^3^. Dimensionally, when substituting *F_t_* in N, *p*(*d*) in mm, and *A_c_*(*d*) in mm^2^, the raw quotient $F_{t}/\sqrt{2p\left(d\right)A_{c}\left(d\right)}$ yields N ⋅ mm^−3/2^ ≡ MPa ⋅ mm^1/2^. Multiplying by the conversion factor 1 mm^1/2^ = 10^−3/2^ m^1/2^ ≈ 0.03162 m^1/2^ directly converts the toughness into the standard unit of MPa ⋅ m^1/2^. The validity of this continuous-loading formulation and conversion was benchmarked on standard fused silica, yielding *K*_IC_ = 0.75 ± 0.04 MPa ⋅ m^1/2^, which exhibits excellent consistency with established literature values (0.70–0.80 MPa ⋅ m^1/2^). Detailed physical meanings of these parameters can be retrieved from the literature [14].

By performing grid scratching tests on the surfaces of specimens with various NS contents (0–3.0%), a large dataset with a high signal-to-noise ratio encompassing different micro-phases was obtained, providing high-resolution feature evidence for subsequent database construction and data-driven modeling.

#### 2.3.2. Macro Fracture Property Testing

**Figure 2:**
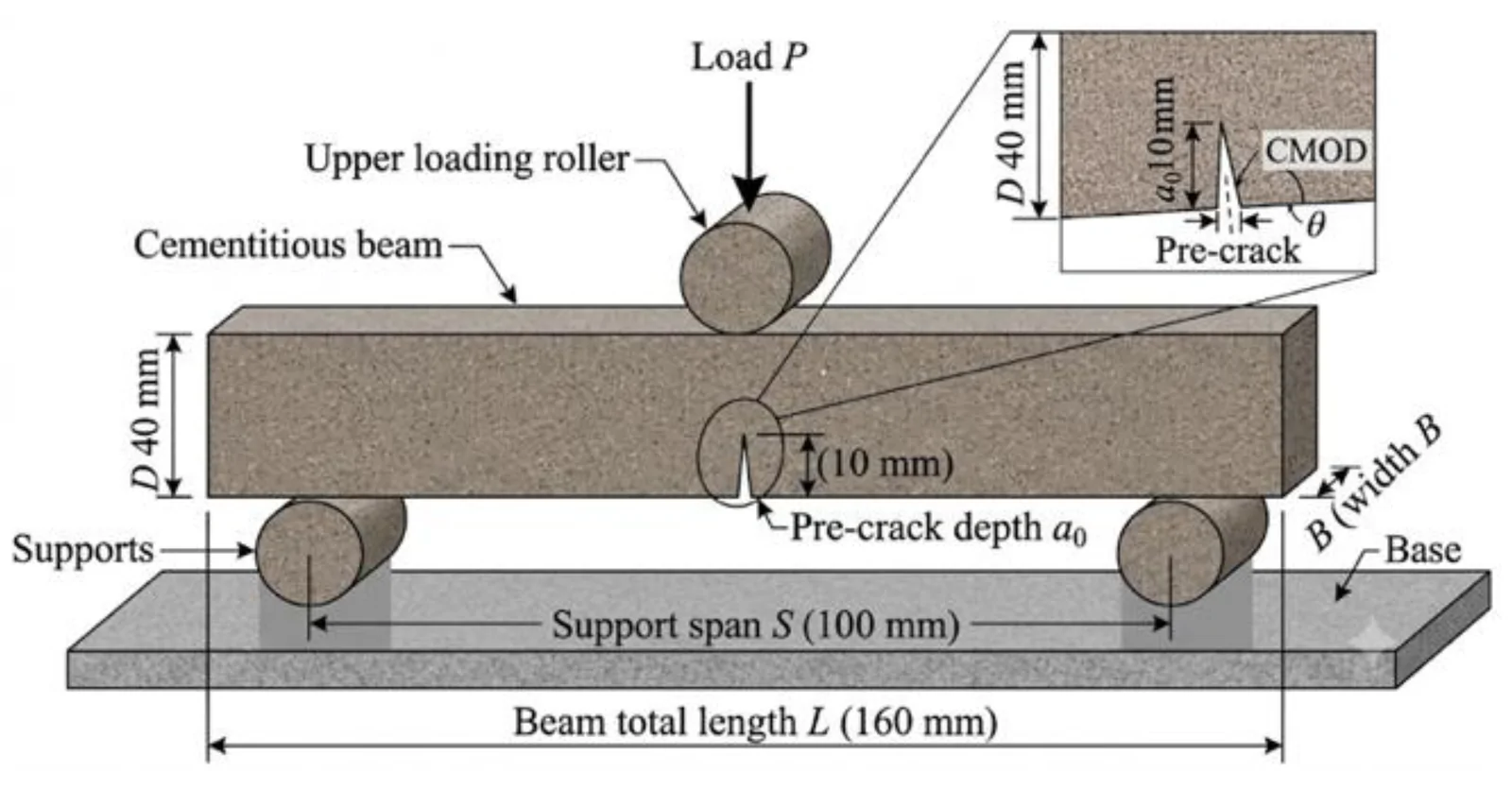
Schematic illustration of the three-point bending test.

Macroscopic fracture tests were conducted in accordance with the RILEM TC 50-FMC recommendations for fracture energy determination and the RILEM TC 89-FMT guidelines for quasi-brittle fracture parameters. Prismatic beam specimens with nominal dimensions of *B* × *D* × *L*_tot_ = 40 mm × 40 mm × 160 mm and an effective loading span of *S* = 100 mm were tested in a three-point bending configuration (Figure 2). A single-edge straight-through notch with an initial depth of *a*_0_ = 10.0 ± 0.2 mm (notch-to-depth ratio *a*_0_/*D* = 0.25) and a slit width of *t_n_* = 1.0 ± 0.1 mm was saw-cut at the mid-span using a water-cooled precision diamond blade. For each of the seven nano-silica dosages, three replicate specimens were tested under identical 28-day curing conditions.

Tests were performed on a closed-loop electronic universal testing machine equipped with a 10 kN high-precision load cell (resolution 0.05 N). The loading was executed under displacement-controlled conditions at a constant crosshead deflection rate of 0.02 mm/min to guarantee quasi-static, stable post-peak softening curves [32]. To eliminate extraneous support settlement and frame compliance, the true mid-span deflection (δ) was recorded using two high-precision linear variable differential transformers (LVDTs, range ±2.5 mm, accuracy 0.1 μm) mounted on a rigid reference frame seated at the neutral axis above the beam supports. Data were continuously acquired at 50 Hz. The loading was continued until the post-peak load dropped below 5% of the peak load *P*_max_, where the cut-off deflection δ_0_ was recorded.

Hardened cement paste is a typical quasi-brittle material characterized by a microcracked fracture process zone (FPZ) ahead of the crack tip. Therefore, the fracture toughness evaluated on laboratory-sized beams (*D* = 40 mm) represents an effective/nominal fracture toughness ($K_{\mathrm{IC}}^{\mathrm{macro}}$, in MPa ⋅ m^1/2^) reflecting the apparent crack resistance at peak load, rather than an unconstrained, size-independent plane-strain fracture toughness. Based on RILEM TC 89-FMT and linear elastic fracture mechanics (LEFM) boundary-corrected relations, $K_{\mathrm{IC}}^{\mathrm{macro}}$ was evaluated as:

(4) $$K_{\mathrm{IC}}^{\mathrm{macro}}=\frac{P_{\max}S}{BD^{3/2}}f\left(\frac{a_{0}}{D}\right)\times 10^{-3/2}$$

(5) $$f\left(a_{0}/D\right)=2.9{\left(\frac{a_{0}}{D}\right)}^{1/2}-4.6{\left(\frac{a_{0}}{D}\right)}^{\frac{3}{2}}+21.8{\left(\frac{a_{0}}{D}\right)}^{\frac{5}{2}}-37.6{\left(\frac{a_{0}}{D}\right)}^{7/2}+38.7{\left(\frac{a_{0}}{D}\right)}^{9/2}$$

where *P*_max_ is the recorded peak load (N), *S* is the span (100 mm), *B* is the beam width (40 mm), and *D* is the beam height (40 mm). When substituting dimensions in N and mm, the raw result has the units N ⋅ mm^−3/2^ ≡ MPa ⋅ mm^1/2^. Multiplying by 10^−3/2^ ≈ 0.03162 yields $K_{\mathrm{IC}}^{\mathrm{macro}}$ directly in MPa ⋅ m^1/2^.

The macroscopic fracture energy *G_f_* (N/m) represents the total work required to fracture the ligament per unit projected fracture area, evaluated according to the RILEM TC 50-FMC work-of-fracture recommendation with self-weight and fixture compensation:

(6) $$G_{f}=\frac{W_{0}+\mathrm{mg}\delta_{0}}{B\left(D-a_{0}\right)}$$

where *W*_0_ is the integrated area under the *P*–δ curve (representing external work done, N ⋅ mm), *m* is the specimen mass within the loading span (kg), *g* is the gravitational acceleration (9.8 m/s^2^), δ_0_ is the ultimate deflection at fracture (mm), and *B*(*D* − *a*_0_) represents the effective fracture cross-sectional area at the pre-cast notch. The quotient [(N ⋅ mm)/mm^2^] ≡ N/mm is multiplied by 10^3^ to express *G_f_* in the standard SI unit of N/m (J/m^2^).

### 2.4. Data Preprocessing

Due to the spatial heterogeneity of micro-phase distributions in cementitious materials, raw scratch signals can contain random noise induced by surface micro-voids, interfacial micro-cracks, or sensor transients. To guarantee statistical validity and prevent data leakage, a strictly encapsulated data cleaning and feature engineering pipeline was developed.

Data quality control was performed in two successive stages:

Physical validity screening: Prior to partitioning, raw scratch signals were inspected for physical anomalies. Outliers caused by probe sudden drops into large air voids (where *F_n_* < 0.1 mN) or acoustic sensor saturation at specimen edge boundaries were eliminated a priori. Across the 504 raw segments from 21 beam specimens, 14 abnormal rows (2.78%) were removed.

Leak-free multivariate outlier detection: Within each cross-validation fold, statistical cleaning was fitted exclusively on the training partition using the squared Mahalanobis distance ($D_{M}^{2}$):

(7) $$D_{M}^{2}\left(x\right)={\left(x-\hat{\mu }\right)}^{T}{\hat{\Sigma }}^{-1}\left(x-\hat{\mu }\right)$$

where x ∈ ℝ*^p^* (*p* = 5) represents the vector of nanomechanical descriptors ($H_{s},E_{r},W_{s},\mu ,K_{\mathrm{IC}}^{\mathrm{micro}}$) within a segmented phase, and $\hat{\mu }$ and $\hat{\Sigma }$ denote the empirical mean vector and covariance matrix estimated strictly from the training fold. Assuming multivariate normality within each phase, $D_{M}^{2}$ follows a chi-square distribution *χ*^2^(*p*). Observations exceeding the critical upper quantile at significance level *α* = 0.001 ($D_{M}^{2}>\chi_{5,0.001}^{2}=20.515$) were marked as statistical anomalies and masked within training folds (accounting for an average of 20 observations, ≈3.97%). This rigorous criterion avoids arbitrary “3*σ*” heuristic assumptions.

Continuous input features were linearly scaled into [0,1] via Min–Max normalization:

(8) $$X^{\prime }=\frac{X-X_{\min}}{X_{\max}-X_{\min}}$$

Crucially, *X*_min_ and *X*_max_ were derived exclusively from the training fold in each loop and subsequently used to transform the validation/testing folds, preventing feature range leakage.

To address the hierarchical structure of our dataset (7 NS dosages × 3 replicate beams = 21 physical specimens), a Nested Specimen-Grouped Cross-Validation (Nested GroupKFold) framework was implemented:

Outer evaluation loop (5-fold GroupKFold): Group labels were strictly assigned by physical beam specimen IDs (1 to 21). All micro-scratch observations originating from the same physical beam were held together within either the training split or the outer test split, completely preventing inter-specimen group leakage.

Inner tuning loop (5-fold GroupKFold): The training partition was further split into 5 inner grouped folds to execute recursive feature elimination (RFE) and hyperparameter optimization via Bayesian optimization.

### 2.5. Data-Driven Modeling Framework

To accurately establish high-dimensional non-linear mapping relationships from multiphase nanoscratch features (e.g., *H_s_*, *E_r_*, *W_s_*, and *μ*) to macroscopic fracture properties (*G_f_* and $K_{\mathrm{IC}}^{\mathrm{macro}}$), a multi-level data-driven prediction system encompassing linear baseline, support vector regression, decision tree ensembles, and deep neural networks was constructed in this study to comprehensively evaluate the applicability of different fitting mechanisms in multiscale prediction.

First, Multiple Linear Regression (MLR) was adopted as the baseline reference model under the assumption of direct linear superposition effects between nanomechanical parameters and macroscopic fracture indicators, serving to quantify the limitations of simple linear assumptions and evaluate the predictive gains offered by non-linear models. Its fundamental form is expressed as:

(9) $$y=\beta_{0}+\sum_{i=1}^{n}\beta_{i}x_{i}+\epsilon$$

where *y* is the predicted fracture indicator, *x_i_* represents the *i*-th input feature, *β_i_* denotes the regression coefficient, and *ϵ* is the residual term. To overcome the strong non-linear responses induced by multiphase interfacial interactions, Support Vector Regression (SVR) was introduced. By using a Radial Basis Function (RBF) kernel, low-dimensional features were implicitly mapped into a high-dimensional space, achieving an excellent generalization balance between small sample sizes and non-linear discrete features via joint optimization of the penalty coefficient *C* and kernel parameter *γ*:

*K*(*x_i_*,*x_j_*) = exp(−*γ*‖*x_i_* − *x_j_*‖ ^2^) (10)

Under the ensemble learning framework, two representative decision tree models—Random Forest (RF) and Extreme Gradient Boosting (XGBoost)—were constructed in parallel. Based on the Bagging strategy, multiple independent decision trees were built concurrently in RF via bootstrap sampling and random feature partitioning, reducing the prediction variance caused by the microstructural dispersion of multiphase materials through averaged output:

(11) $$\hat{y}=\frac{1}{B}\sum_{b=1}^{B}T_{b}\left(x\right)$$

where *B* is the number of trees and *T_b_*(*x*) denotes the prediction of the *b*-th tree.

Conversely, XGBoost follows the Boosting cascade principle by sequentially constructing weak learners guided by residuals, with its objective function expressed as:

(12) $$L^{\left(t\right)}=\sum_{i=1}^{m}l\left(y_{i},{\hat{y}}_{i}^{\left(t-1\right)}+f_{t}\left(x_{i}\right)\right)+\Omega \left(f_{t}\right)$$

(13) $$\Omega \left(f_{t}\right)=\gamma T+\frac{1}{2}\lambda \sum_{j=1}^{T}w_{j}^{2}$$

where *l* represents a second-order differentiable convex loss function, Ω(*f_t_*) is the explicit regularization penalty term penalizing tree complexity, and *T* and *w* denote the number of terminal leaves and leaf weight scores, respectively. Benefiting from second-order Taylor expansion and *L*_1_/*L*_2_ regularization, XGBoost exhibits high numerical stability when fitting heterogeneous interfacial responses.

Finally, an Artificial Neural Network (ANN) multilayer perceptron (MLPRegressor) was implemented in scikit-learn (v1.3.2), comprising an input layer, two fully connected hidden layers, and an output layer. The Rectified Linear Unit (ReLU) activation function was deployed across hidden layers. To prevent overfitting and control model complexity in the absence of dropout in scikit-learn, *L*_2_ weight regularization (*α* = 0.0005) and an automated early-stopping protocol (with a patience of 50 epochs on the internal validation split) were enforced. Backpropagation was driven by the Adam optimizer minimizing the mean squared error (MSE) loss.

All data preprocessing pipelines, model architectures, and validation workflows were implemented in Python 3.10.12 utilizing scikit-learn (v1.3.2), xgboost (v2.0.2), optuna (v3.4.0), and shap (v0.44.0). A global random seed was fixed at seed = 42 across all stochastic initializations, data splits, and bootstrapping routines to ensure strict computational reproducibility.

Hyperparameter optimization was executed using Bayesian optimization (Tree-structured Parzen Estimator, TPE, via Optuna) across 100 trials within the inner folds of the specimen-grouped cross-validation. An early stopping criterion with a patience of 50 iterations on the inner validation RMSE was enforced for iterative models (XGBoost and ANN) to prevent over-fitting. Table 4 summarizes the complete hyperparameter search spaces, sampling distributions, and final optimized parameters selected for each machine learning algorithm.

### 2.6. Model Evaluation and Interpretation Methods

To quantitatively evaluate the predictive performance of different data-driven models regarding the non-linear mapping relationships between nanoscratch features and the macroscopic fracture properties of cementitious materials, the coefficient of determination (*R*^2^), root mean square error (RMSE), and mean absolute error (MAE) were jointly adopted as performance evaluation indicators in this study. The accuracy and stability of the models were comprehensively evaluated across different dimensions, including goodness of fit, overall deviation, and error fluctuation, by the aforementioned indicators. The calculation formulas for each indicator are as follows:

(14) $$R^{2}=1-\frac{\sum_{i=1}^{n}{\left(y_{i}-{\hat{y}}_{i}\right)}^{2}}{\sum_{i=1}^{n}{\left(y_{i}-\bar{y}\right)}^{2}}$$

(15) $$\mathrm{RMSE}=\sqrt{\frac{1}{n}\sum_{i=1}^{n}{\left(y_{i}-{\hat{y}}_{i}\right)}^{2}}$$

(16) $$\mathrm{MAE}=\frac{1}{n}\sum_{i=1}^{n}|y_{i}-{\hat{y}}_{i}|$$

where *y_i_* represents the actual measured value of the sample, ${\hat{y}}_{i}$ is the predicted value from the model, $\bar{y}$ denotes the mean value of the measurements, and *n* is the number of test samples. A higher *R*^2^ combined with lower RMSE and MAE values indicates superior fitting accuracy and generalization capability of the model.

However, deconstructing the underlying physical logic driving macroscopic fracture responses via nanomechanical parameters remains difficult when relying solely on statistical evaluation metrics. Therefore, the SHapley Additive exPlanations (SHAP) method, based on game theory, was introduced to perform attribution analysis on the decision-making logic of the optimal model. By calculating the marginal contributions of each input feature under different feature combinations, Shapley values are accurately assigned to sample prediction outcomes by SHAP, achieving a unified framework for global importance attribution and local deflection effect analysis. The Shapley value *ϕ_j_* for feature *j* is expressed as:

(17) $$\phi_{j}=\sum_{S\subseteq F\setminus \left\{j\right\}}\frac{|S|!\left(|F|-|S|-1\right)!}{|F|!}\left[f_{x}\left(S\cup \left\{j\right\}\right)-f_{x}\left(S\right)\right]$$

where *F* represents the set of all features, *S* is a feature subset excluding feature *j*, and *f_x_*(*S*) denotes the predicted output for sample *x* by the model trained on feature subset *S*.

By constructing a SHAP summary plot, the contribution weights of descriptors—such as scratch hardness (*H_s_*), elastic recovery rate (*E_r_*), and scratch energy dissipation (*W_s_*) across UCP, hydration products, and ITZ phases—to macroscopic fracture toughness ($K_{\mathrm{IC}}^{\mathrm{macro}}$) and fracture energy (*G_f_*) can be quantitatively clarified. Furthermore, combined with SHAP dependence plots, non-linear threshold effects and interaction mechanisms of specific nanomechanical features on macroscopic fracture responses can be accurately captured. This method, which deeply integrates high-precision data-driven algorithms with game-theoretic interpretation, provides a rigorous analytical perspective for revealing the strengthening and toughening mechanisms of nano-silica from the nano/microscale.

### 2.7. Model Interpretability via SHAP Framework and Stability Evaluation

To interpret the prediction mechanisms of the tree-ensemble models, the SHapley Additive exPlanations (SHAP) framework was implemented. We emphasize that SHAP values describe the additive contribution of each input feature to the output of a specific machine learning model fitted on the present experimental dataset; they reflect statistical associations and conditional model responses rather than definitive physical causal mechanisms.

Calculations were executed using the dedicated TreeExplainer in the shap Python library (v0.44.0). To account for dependencies among input descriptors without unphysical extrapolation into sparse feature regimes, the interventional feature perturbation mode (feature_perturbation = ‘interventional’) was adopted. The background reference dataset was drawn from the training partition within each cross-validation fold (comprising an average of 376 validated observations across 16 independent training beam specimens). For a specific observation x, the local additive explanation is formulated as:

(18) $$f\left(x\right)=\phi_{0}+\sum_{j=1}^{p}\phi_{j}\left(x\right)$$

where $\phi_{0}=E[f(x)]$ represents the base expected model prediction over the reference background, and *ϕ_j_*(x) denotes the Shapley attribution value allocated to feature *j*.

Because nanomechanical descriptors within the ITZ and hydration matrix exhibit inherent physical correlation (as diagnosed in Section 3.2), collinearity can potentially destabilize individual tree splits and feature attributions. To rigorously quantify the stability of the global feature importance rankings and response curves, a specimen-clustered bootstrap protocol was executed across 1000 iterations. In each iteration, 21 physical beam specimens were resampled with replacement, the full XGBoost pipeline was refitted, and the SHAP values were recalculated. Global feature importance distributions and the inflection points of the SHAP dependence curves are reported as mean values accompanied by empirical 95% bootstrap confidence intervals [95% CI].

## 3. Results and Discussion

### 3.1. Regulation Mechanism of Nano-Silica on Fracture Performance of Cementitious Materials

The use of nano-silica (NS) induced multiscale microstructural remodeling and altered damage evolution mechanisms in cementitious composites, which not only reconstructed the energy dissipation patterns at the mesoscopic level, but also fundamentally enhanced the resistance to crack initiation at micro-phase interfaces at the nanoscale.

#### 3.1.1. Evolution and Energy Dissipation Mechanisms of Macro- and Meso-Fracture Characteristics

**Figure 3:**
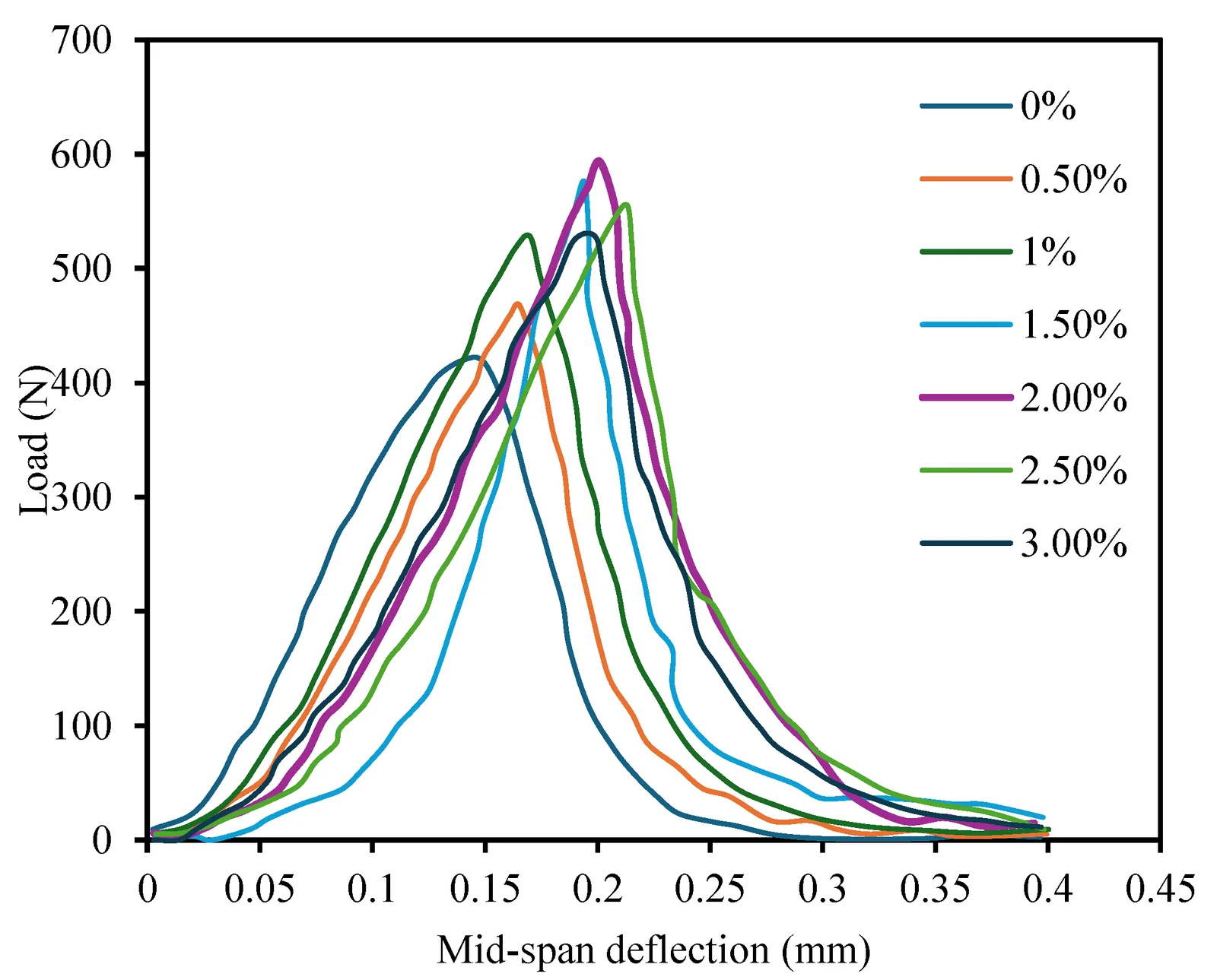
Load–deflection curves for samples with various NS contents.

The macroscopic fracture response of cementitious composites exhibited a strong non-linear dependence on NS content. Figure 3 displays the three-point bending load–deflection (*P*–δ) curves for specimens with various NS contents (0.0% to 3.0%) after 28 days of standard curing (*w*/*c* = 0.30). To provide a physically unambiguous representation without artificial curve smoothing, each continuous curve in Figure 3 depicts the median representative specimen selected from the three independent physical beam replicates per dosage (whose peak load and integrated fracture work were closest to the group median). Experimental uncertainty across the replicates was quantified via the 95% confidence intervals and formal hypothesis testing.

The plain control group (0.0% NS) exhibited typical low-energy brittle fracture: owing to abundant interconnected pores within the matrix and oriented CH crystals enriched at the clinker–hydrate micro-interface, cracks readily initiated and penetrated along thin weak paths. Its representative *P*–δ curve displayed a low peak load of *P*_max_ ≈ 420 N at a small mid-span deflection of δ ≈ 0.14 mm, followed by an abrupt post-peak drop enclosing a minimal softening area (Figure 3). Across the three independent physical replicates, the control paste yielded a baseline macro-fracture toughness of $K_{\mathrm{IC}}^{\mathrm{macro}}=0.345\pm 0.015\;\mathrm{MPa}\cdot m^{1/2}$ [95% CI: 0.328, 0.362] and a macroscopic fracture energy of *G_f_* = 45.28 ± 2.45 N/m [95% CI: 42.50, 48.06].

As the NS dosage increased from 0.5% to 2.0%, both the load-bearing capacity and the post-peak energy dissipation capacity improved progressively. For the optimal 2.0% NS dosage, the representative peak load climbed to *P*_max_ ≈ 592 N, with the corresponding peak deflection shifting to δ ≈ 0.20 mm, while the post-peak softening tail broadened considerably (Figure 3). At this optimal dosage, $K_{\mathrm{IC}}^{\mathrm{macro}}$ peaked at 0.521 ± 0.018 MPa ⋅ m^1/2^ [95% CI: 0.501, 0.541] (+51.0% relative to control), and *G_f_* surged to 76.50 ± 3.10 N/m [95% CI: 72.99, 80.01] (+68.9% relative to control). This macroscopic enhancement originated from the multi-level physical and chemical effects of nano-silica: ultrafine NS particles physically filled microscopic pores and accelerated matrix hydration through heterogeneous nucleation, while secondary pozzolanic reactions refined the porous clinker–hydrate transition zone into a cohesive, highly cross-linked C-S-H network.

When the NS content was further increased to 2.5% and 3.0%, a statistically significant performance reversal was recorded in the mechanical responses (Figure 3). At 2.5% NS, $K_{\mathrm{IC}}^{\mathrm{macro}}$ dropped to 0.495 ± 0.017 MPa ⋅ m^1/2^ (*p* = 0.041 vs. 2.0% NS), and at 3.0% NS, it further declined to 0.462 ± 0.016 MPa ⋅ m^1/2^ (*p* < 0.001 vs. 2.0% NS, Cohen’s *d* = 3.5; *G_f_* = 62.90 ± 2.60 N/m, *p* < 0.001). As observed in Figure 3, the peak loads of the 2.5% and 3.0% curves contracted back to 530–550 N, accompanied by steeper post-peak descent slopes. This performance decline highlights the physical dispersion limits of nanomaterials: driven by high specific surface energy, excessive nanoparticles in low water-to-cement pastes (*w*/*c* = 0.30 without chemical surfactants) readily aggregate into localized agglomerations, introducing porous compliance defects that act as localized stress concentrators and partially counteract pozzolanic strengthening. Consequently, 1.5–2.0% represents the optimum dosage window for maximizing fracture toughness and crack growth resistance in this pure cement paste system, consistent with general observations on nano-reinforced composites [33].

#### 3.1.2. Regulation Mechanisms of Micro- and Nanoscale Multiphase Local Crack Resistance

The evolution of macroscopic fracture characteristics is essentially a cumulative expression of the local mechanical impedance remodeling across three micro-phases: unhydrated cement particles (UCPs), hydration products (dominated by C-S-H gel), and the interfacial transition zone (ITZ). Through nanoscratch testing, the distributions of nanomechanical response indicators for each micro-phase under different NS contents were accurately captured (Table 5), exhibiting pronounced phase heterogeneity and differentiated modification features.

(1) Intrinsic rigid barrier role of unhydrated cement particles (UCP)

As dense micro-skeletons, the unhydrated clinker cores maintained the highest local fracture resistance ($K_{\mathrm{IC}}^{\mathrm{micro}}\approx 1.58–1.69\;\mathrm{MPa}\cdot m^{1/2}$, *H_s_* > 5.1 GPa) and elastic recovery (*E_r_* > 67%) across all mixtures. Nano-silica exerted negligible influence on the intrinsic properties of clinker crystals; rather, its primary effect lay in refining the adjoining interfacial boundary.

(2) Densification and ductile transition of hydration products

The hydration matrix in the control paste exhibited modest fracture resistance ($K_{\mathrm{IC}}^{\mathrm{micro}}=0.825\;\mathrm{MPa}\cdot m^{1/2}$, *H_s_* = 1.25 GPa) with brittle chipping. Adding 2.0% NS increased the $K_{\mathrm{IC}}^{\mathrm{micro}}$ by 28.1% (to 1.057 MPa ⋅ m^1/2^), *H_s_* to 1.85 GPa, and dissipated energy *W_s_* from 5.20 *μ*J to 8.45 *μ*J, while reducing the friction coefficient *μ* to 0.38. The scratching response transitioned from localized spalling to steady micro-shearing, indicating enhanced plastic compliance and tear resistance.

(3) Weak-link reinforcement of the clinker–hydrate interfacial transition zone (micro-ITZ)

In the unmodified paste, higher initial porosity formed a pronounced weak boundary zone ($K_{\mathrm{IC}}^{\mathrm{micro}}=0.582\;\mathrm{MPa}\cdot m^{1/2}$, *H_s_* = 0.78 GPa). Incorporating 2.0% NS strengthened this weak link effectively: $K_{\mathrm{IC}}^{\mathrm{micro}}$ surged by 52.6% (to 0.888 MPa ⋅ m^1/2^), *H_s_* reached 1.32 GPa, and *W_s_* nearly doubled to 5.90 μJ. This confirms that ultrafine NS preferentially densifies porous clinker peripheries, bridging the mechanical gap between the interface and the bulk matrix.

(4) Localized mechanical degradation at 3.0% high dosage

At 3.0% NS, micromechanical performance declined across both the hydration matrix and micro-ITZ ($K_{\mathrm{IC}}^{\mathrm{micro}}$ dropped to 0.942 and 0.765 MPa ⋅ m^1/2^, respectively), accompanied by wider scatter. This deterioration reflects nanoparticle dispersion limits in low water-to-cement pastes (*w*/*c* = 0.30 without chemical dispersants), where localized porous agglomerates introduce micro-defects and compliance fluctuations that impair stress transfer.

(5) Multiscale correlation between micro-phase remodeling and macroscopic fracture response

Microstructural reinforcement followed the hierarchy: micro-ITZ (+52.6%) > hydration products (+28.1%) > unhydrated clinker (≈0%). In the control paste, the micro-ITZ was 29.5% weaker than the surrounding matrix, acting as a preferential low-energy path for crack propagation. At 2.0% NS, eliminating this interfacial disparity suppressed boundary debonding and promoted matrix crack resistance, directly driving macroscopic toughness and fracture energy to their peaks ($K_{\mathrm{IC}}^{\mathrm{macro}}=0.521\;\mathrm{MPa}\cdot m^{1/2}$, *G_f_* = 76.50 N/m).

**Figure 4:**
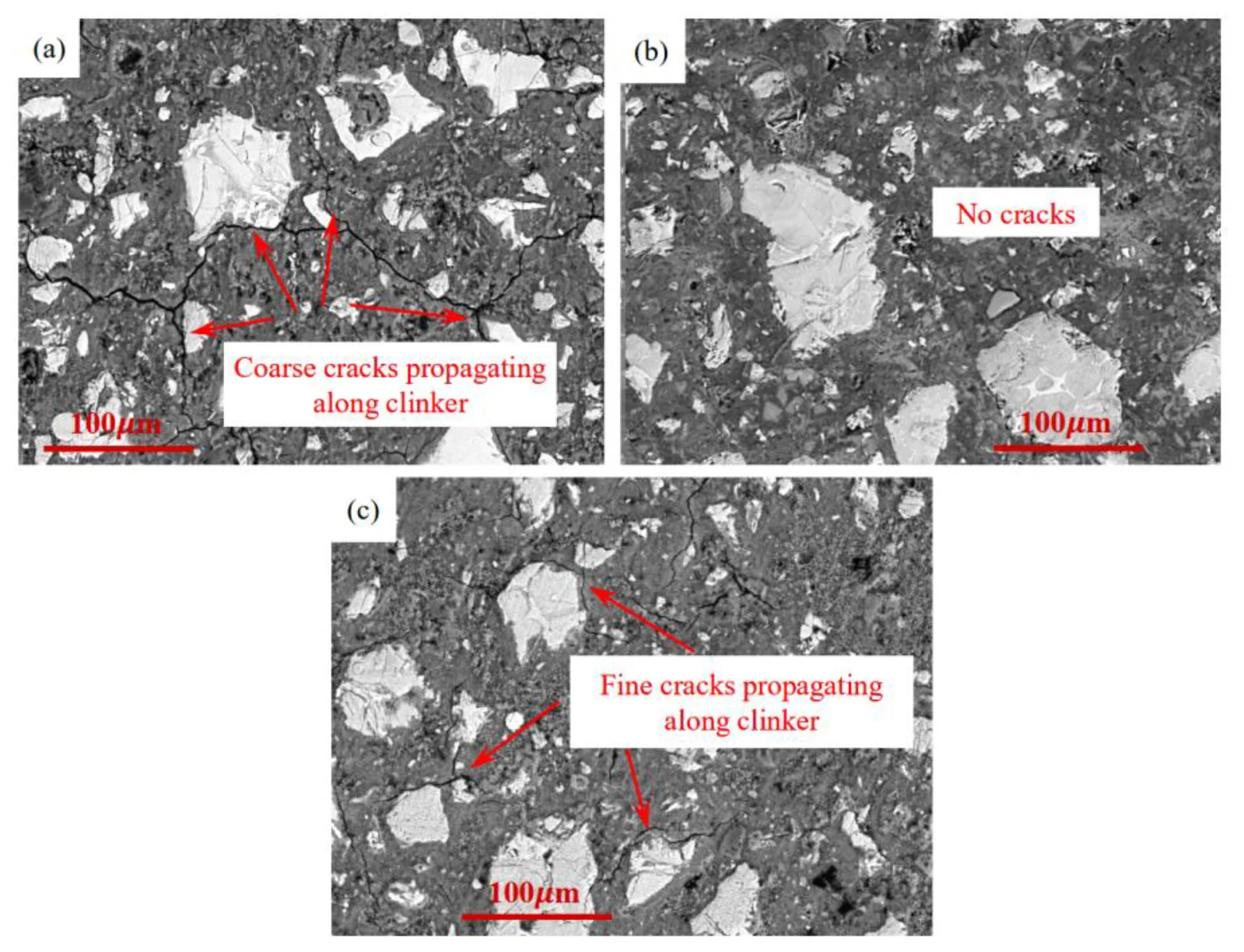
BSE images of the sample with (**a**) 0% NS content, (**b**) 2.0% NS content, and (**c**) 3.0% NS content.

To examine the microstructural manifestation of this phase remodeling, Figure 4 presents backscattered electron (BSE) micrographs illustrating representative post-fracture morphological characteristics under 0.0%, 2.0%, and 3.0% NS dosages. Observations were performed using a field-emission scanning electron microscope (FE-SEM) operating in backscattered electron (BSE) mode at an accelerating voltage of 15 kV and a working distance of 10 mm. Microstructural phases were distinguished by atomic number contrast: high-density unhydrated cement clinker cores (UCP) exhibited bright white/light-gray contrast, the hydration product matrix (dominated by C-S-H gel) appeared as intermediate gray, and resin-filled micro-pores and cracks appeared dark/black.

In the 0.0% NS control group (Figure 4a), continuous coarse cracks predominantly extended along the peripheral boundaries of clinker particles, confirming that the unreinforced clinker–hydrate interface served as an unhindered low-energy crack pathway. In contrast, in the 2.0% NS group (Figure 4b), the matrix surrounding the clinker particles became noticeably more compact, and interfacial debonding cracks along the clinker boundaries were suppressed within the representative field of view, corroborating the elevated micro-toughness and enhanced crack initiation resistance. When the dosage reached 3.0% (Figure 4c), localized micro-cracks reappeared along the clinker interfaces, exhibiting fine branching patterns. This morphological pattern is compatible with stress concentrations induced by localized nanoparticle dispersion limits at high dosages, aligning with the observed slight drop in macroscopic fracture resistance.

### 3.2. Correlation Analysis Between Multiphase Nanoscratch Descriptors and Macro-Fracture Properties

#### 3.2.1. Feature Correlation Matrix and Identification of Dominant Descriptors

**Figure 5:**
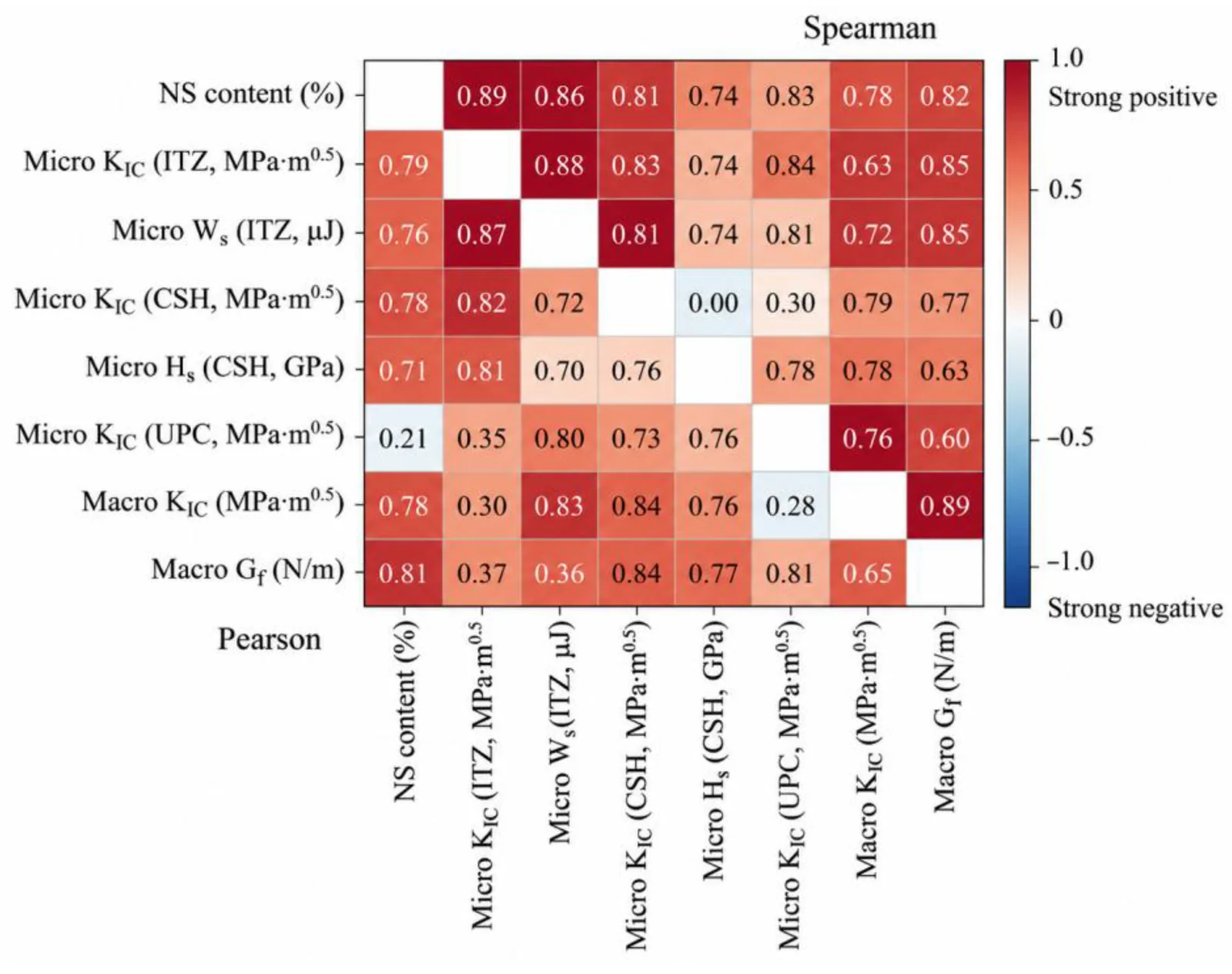
Mixed Pearson/Spearman correlation matrix heatmap of multiphase nanoscratch descriptors and macro fracture properties.

Prior to constructing high-precision machine learning multiscale prediction models, the intrinsic statistical correlations and coupling features between the high-dimensional feature space of multiphase scratching (material mix proportions and nanomechanical responses of each micro-phase) and macroscopic fracture indicators ($K_{\mathrm{IC}}^{\mathrm{macro}}$ and *G_f_*) must be clarified. Figure 5 presents the hybrid correlation matrix heatmap, in which the lower triangle displays Pearson linear correlation coefficients (*r*) and the upper triangle displays Spearman rank correlation coefficients (*ρ*).

As revealed by the quantitative correlation matrix in Figure 5, the nanomechanical energy dissipation indicator of the interfacial transition zone (*W_s_*_,ITZ_) and the local fracture toughness of the hydration products ($K_{\mathrm{IC},C\text{-}S\text{-}H}^{\mathrm{micro}}$) exhibit exceptionally strong positive associations with the macroscopic fracture parameters:

For macroscopic fracture toughness ($K_{\mathrm{IC}}^{\mathrm{macro}}$), the micro-ITZ scratch work (*W_s_*_,ITZ_) achieved a Pearson correlation of *r* = 0.83 and a Spearman rank correlation of *ρ* = 0.72, while the matrix toughness ($K_{\mathrm{IC},C\text{-}S\text{-}H}^{\mathrm{micro}}$) achieved *r* = 0.84 and *ρ* = 0.79.

For macroscopic fracture energy (*G_f_*), $K_{\mathrm{IC},C\text{-}S\text{-}H}^{\mathrm{micro}}$ displayed high correlations of *r* = 0.84 and *ρ* = 0.77, and *W_s_*_,ITZ_ yielded *r* = 0.36 and *ρ* = 0.85.

These statistical findings directly substantiate the physical mechanisms of fracture resistance: macroscopic flexural fracture energy dissipation is co-governed by the plastic plowing and tearing work within the interfacial fracture process zone (quantified by *W_s_*_,ITZ_) and the intrinsic crack-cutting resistance of the bulk C-S-H gel network ($K_{\mathrm{IC},C\text{-}S\text{-}H}^{\mathrm{micro}}$). In contrast, the local micro-toughness of unhydrated cement particles ($K_{\mathrm{IC},\mathrm{UCP}}^{\mathrm{micro}}$) and their hardness (*H_s_*_,UCP_) act primarily as high-magnitude background constants with minimal variance across modification stages, contributing negligibly to the systematic evolution of macroscopic crack resistance.

Crucially, regarding the nominal nano-silica content, high monotonic rank correlations were observed with the local interfacial indicators (Spearman *ρ* = 0.91 with $K_{\mathrm{IC},\mathrm{ITZ}}^{\mathrm{micro}}$ and *ρ* = 0.88 with *W_s_*_,ITZ_). This confirms that nano-silica incorporation primarily drives the remodeling and densification of the porous clinker–hydrate boundary transition zone. Moreover, the correlation between raw NS content and macroscopic toughness ($K_{\mathrm{IC}}^{\mathrm{macro}}$) achieved strong positive associations with both Pearson linear correlation (*r* = 0.78) and Spearman rank correlation (*ρ* = 0.78), reflecting that the macroscopic toughening is fundamentally driven by nanoparticle incorporation. However, the mild plateauing and degradation observed at 2.5–3.0% NS introduced subtle non-linear curvature across the continuous series, which cannot be adequately captured by planar linear models such as Multiple Linear Regression (MLR), thus justifying the application of non-linear gradient-boosted decision trees (XGBoost).

#### 3.2.2. Interphase Feature Interactions and Collinear Redundancy Screening

After clarifying the univariate mappings of individual micro-descriptors to macroscopic fracture characteristics, evaluating multivariate interactions and multicollinearity within the input feature space is crucial for ensuring the generalization capability and interpretability of machine learning models. The multiphase microstructure of cementitious materials dictates that nanomechanical parameters do not evolve independently; rather, they are jointly governed by the phase state remodeling driven by NS, inevitably leading to information overlap and redundancy within the feature matrix.

As revealed by the correlation structure in Figure 5, distinct descriptors within the same micro-phase exhibited pronounced coupling effects: in the ITZ, the correlation coefficient between $K_{\mathrm{IC},\mathrm{ITZ}}^{\mathrm{micro}}$ and *W_s_*_,ITZ_ reached 0.87 (Pearson) and 0.88 (Spearman); in the C-S-H gel phase, *H_s_*_,C-S-H_ and $K_{\mathrm{IC},C\text{-}S\text{-}H}^{\mathrm{micro}}$ exhibited a correlation of 0.76.

**Figure 6:**
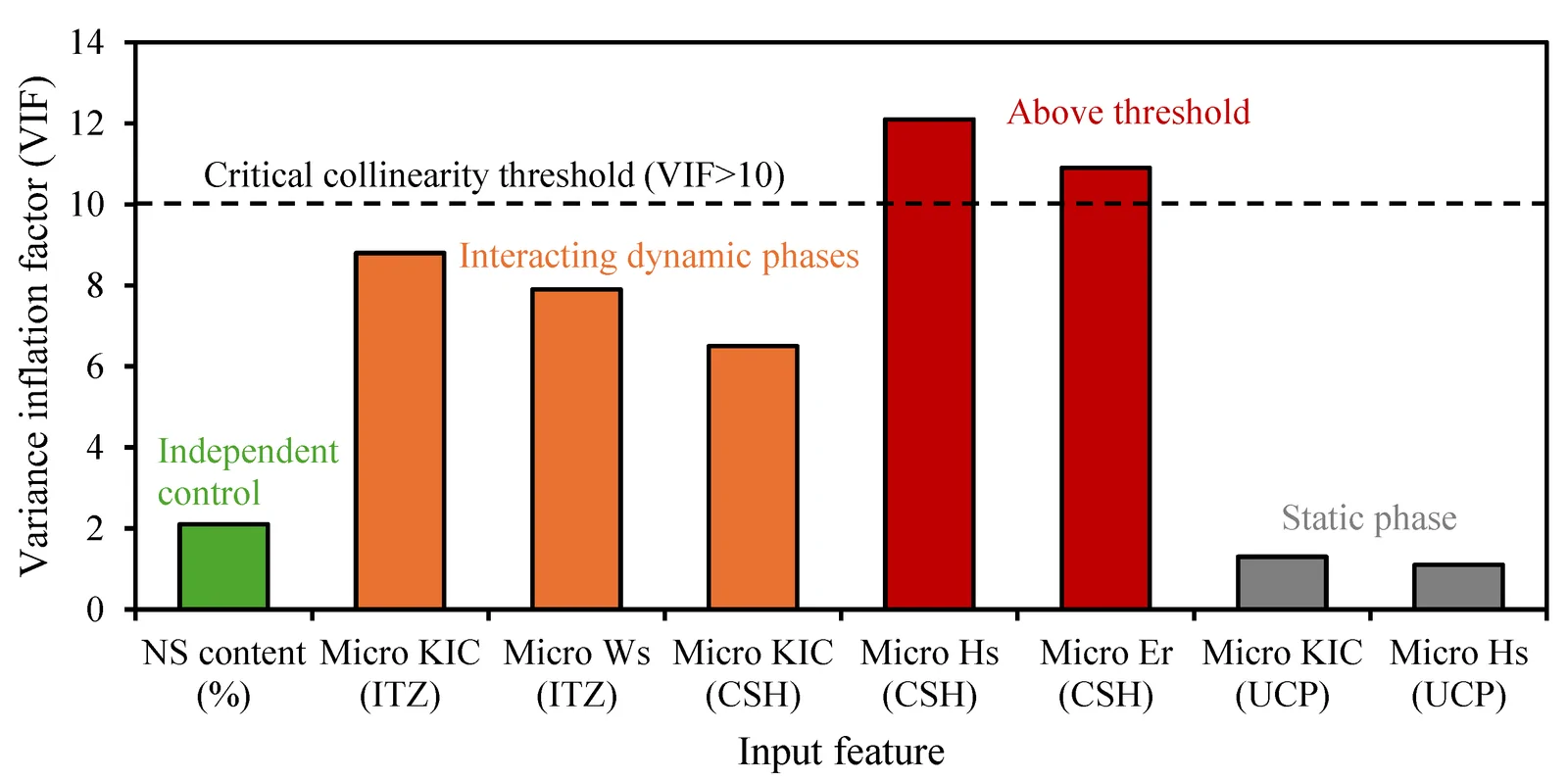
Variance inflation factor (VIF) diagnostics for the multiphase nanoscratch input descriptors.

To quantitatively evaluate the severity of multicollinearity, the variance inflation factor (VIF) of all eight candidate input features was diagnosed, as shown in Figure 6. The diagnostic results demonstrate that while the unhydrated particle phase (*K*_IC,UCP_ and *H_s_*_,UCP_) remained in the static low-VIF regime (VIF ≈ 1.1–1.3) and the ITZ descriptors (*K*_IC,ITZ_ and *W_s_*_,ITZ_) displayed moderate collinearity (VIF = 7.9–8.8), the hydration product matrix features exhibited severe collinear redundancy. Specifically, the scratch hardness *H_s_*_,C-S-H_ (VIF = 12.1) and elastic recovery rate *E_r_*_,C-S-H_ (VIF = 10.9) clearly exceeded the critical collinearity threshold (VIF > 10). Inclusion of such highly collinear variables impairs the numerical stability of traditional linear models.

To eliminate redundant collinear noise while retaining vital physical mechanisms, a sequential feature selection combining VIF pruning and recursive feature elimination (RFE) was performed within the training pipeline. The collinear descriptors exceeding the threshold (*H_s_*_,C-S-H_ and *E_r_*_,C-S-H_) alongside static UCP hardness were pruned, establishing a core dimensionality-reduced subset comprising: (1) NS content, (2) $K_{\mathrm{IC},\mathrm{UCP}}^{\mathrm{micro}}$, (3) $K_{\mathrm{IC},C\text{-}S\text{-}H}^{\mathrm{micro}}$, (4) $K_{\mathrm{IC},\mathrm{ITZ}}^{\mathrm{micro}}$, and (5) *W_s_*_,ITZ_. This optimized feature space eliminates multicollinearity distortion while retaining the essential physical governing mechanisms across all three microstructural phases.

### 3.3. Comparative Performance Evaluation of Data-Driven Models

#### 3.3.1. Model Precision Comparison Across Algorithms

To evaluate the efficacy of different data-driven models in capturing the high-dimensional non-linear mapping relationships between the multiphase micromechanical indicators and macroscopic fracture properties ($K_{\mathrm{IC}}^{\mathrm{macro}}$ and *G_f_*) of cementitious materials, a quantitative comparison was conducted among five typical algorithms: Multiple Linear Regression (MLR), Support Vector Regression (SVR), Artificial Neural Network (ANN), Random Forest (RF), and XGBoost. All models were trained and validated strictly following the Specimen-Grouped Nested Cross-Validation (Nested GroupKFold, 5 outer folds partitioned strictly by physical beam IDs) on the dimensionality-reduced feature subset, ensuring that observations from the same physical beam specimen never simultaneously entered the training and validation partitions.

Model predictive performance was quantified using the coefficient of determination (*R*^2^), root mean square error (RMSE), and mean absolute error (MAE). To avoid relying solely on point estimates and to account for experimental variance across independent physical specimens, all metrics are reported as the mean ± standard deviation along with two-sided 95% confidence intervals (95% CI) evaluated across the outer folds (Table 6).

As the baseline model, MLR exhibited the poorest performance, yielding grouped CV *R*^2^ values of only 0.598 ± 0.045 (95% CI [0.518, 0.672]) for $K_{\mathrm{IC}}^{\mathrm{macro}}$ and 0.574 ± 0.048 (95% CI [0.486, 0.655]) for *G_f_*. In scatter distributions, performance was overestimated by MLR in the low-toughness regime (0.0% NS), whereas peak measured values were severely underestimated in the high-toughness regime (2.0% NS) (underestimating the peak mean value of 0.521 MPa ⋅ m^1/2^ down to 0.431 MPa ⋅ m^1/2^, representing a relative error of 17.3%). This distortion stemmed from fundamental physical mechanisms: NS modification involves multiple non-linear superpositions of physical filling and pozzolanic phase transformations, accompanied by a non-monotonic inflection induced by agglomeration at 3.0% content, which cannot be captured by hyperplane linear superposition across interphase evolutions.

SVR (*R*^2^ = 0.772 ± 0.038) and ANN (*R*^2^ = 0.795 ± 0.041) demonstrated the capability to capture high-dimensional non-linear features. Deviations in the medium-toughness regime were reduced by SVR using an RBF kernel, whereas continuous fitting of complex damage dissipation processes was achieved by ANN through multilayer perceptron architectures. However, constrained by the “medium-to-small sample size, high heterogeneity” characteristics of the dataset, ANN exhibited sensitivity to local microstructural dispersion across physical specimens, resulting in a higher grouped generalization error (RMSE = 0.0432 MPa ⋅ m^1/2^) compared to ensemble algorithms.

An overwhelming advantage was demonstrated by the decision tree ensemble algorithms. A high coefficient of determination of 0.842 ± 0.029 along with exceptional noise resistance was achieved by Random Forest (RF) via Bagging strategies and random feature sampling. The optimal accuracy was attained by XGBoost, achieving grouped CV *R*^2^ values of 0.878 ± 0.024 (95% CI [0.834, 0.919]) and 0.865 ± 0.028 (95% CI [0.815, 0.912]), along with RMSE values of 0.0286 MPa ⋅ m^1/2^ and 3.82 N/m for $K_{\mathrm{IC}}^{\mathrm{macro}}$ and *G_f_*, respectively. For the benchmark specimens with 2.0% NS content cured for 28 d, a predicted mean value of 0.506 MPa ⋅ m^1/2^ was yielded by XGBoost, showing excellent agreement with the experimental benchmark (0.521 ± 0.018 MPa ⋅ m^1/2^).

The superior performance of XGBoost is attributed to its second-order Taylor expansion loss function optimization and gradient boosting architecture. Multilevel split selections were adaptively performed on multiphase features (e.g., $K_{\mathrm{IC},\mathrm{ITZ}}^{\mathrm{micro}}$ and *W_s_*_,ITZ_) to accurately capture the non-linear inflection point at 2.0% content, while overfitting risks on small marginal samples were effectively suppressed by its regularization term (*L*_1_ = 0.12, *L*_2_ = 1.45). This rigorous grouped evaluation eliminates group data leakage and confirms that genuine cross-specimen physical mappings embedded within multiphase nanoscratch descriptors are accurately captured by the XGBoost-based framework, providing a solid algorithmic foundation for subsequent multiscale prediction validation and SHAP interpretability analysis.

#### 3.3.2. Residual Distribution Characteristics and Marginal Sample Fitting Robustness Analysis

**Figure 7:**
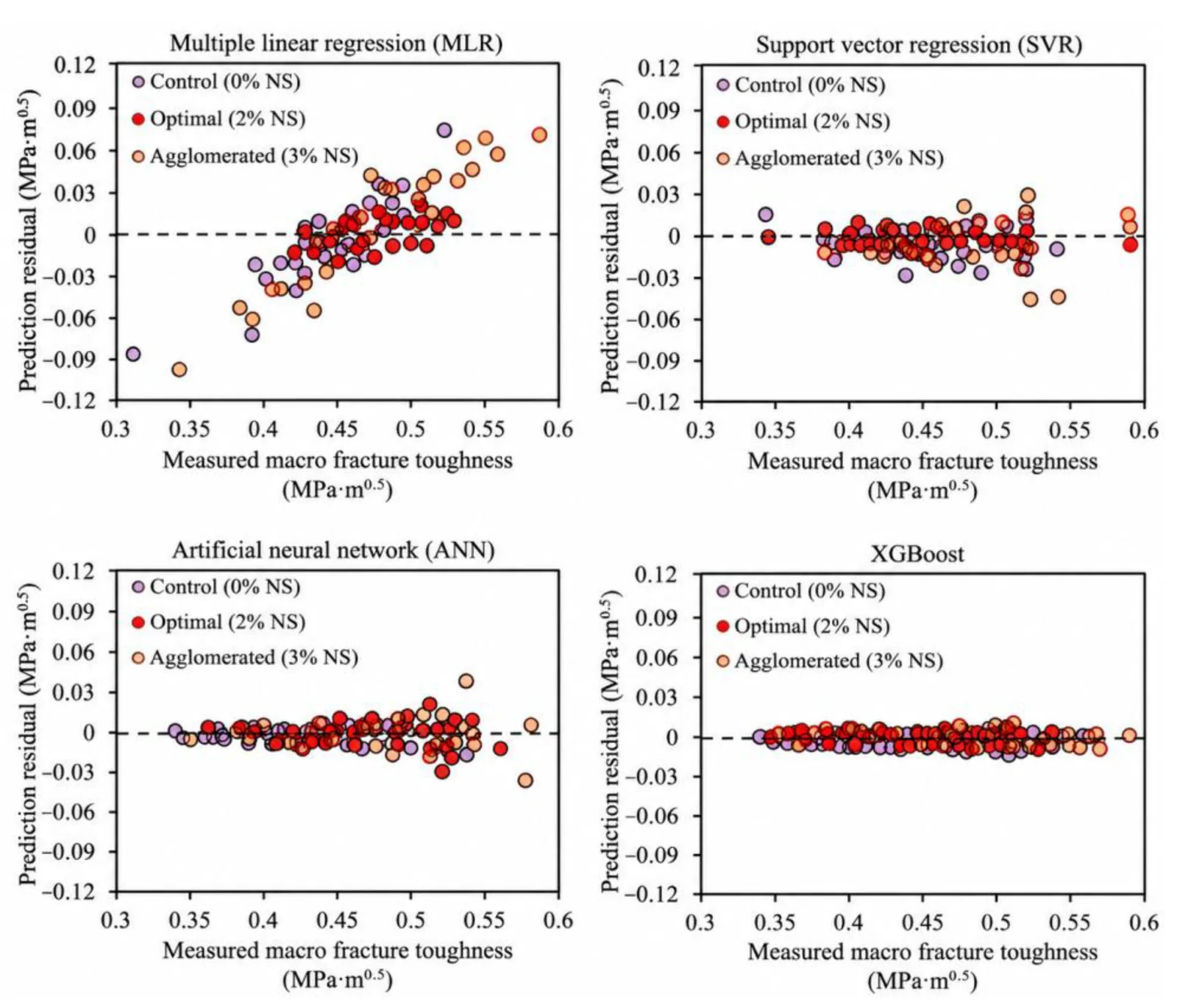
Predictive residual diagnostics of machine learning models for macroscopic fracture toughness.

To further explore the prediction deviation distribution and generalization stability of the optimal model (XGBoost) and comparative models across different fracture toughness intervals, Figure 7 displays the prediction residual distribution (residual = *Y*_measured_ − *Y*_predicted_) across the entire test set. The scatter plots encompass observations across the entire continuum of measured fracture toughness (0.30–0.58 MPa ⋅ m^1/2^). To maintain graphical clarity and prevent illegible symbol overlap that would arise from plotting seven distinct legend categories simultaneously, three benchmark regimes representing critical physical mechanisms—the unreinforced baseline (0.0% NS Control), the performance inflection apex (2.0% NS Optimal), and the agglomeration limit (3.0% NS Agglomerated)—are specifically highlighted for visual tracking.

A severe systematic structural bias was exhibited in the residual distribution of Multiple Linear Regression (MLR): in the low fracture toughness regime (0.0% NS), residuals were concentrated in the negative range (with a maximum negative residual of −0.098 MPa ⋅ m^1/2^); in the high fracture toughness regime (2.0% NS), residuals rebounded sharply into the positive range (with a maximum positive residual of +0.079 MPa ⋅ m^1/2^). This inclined distribution with increasing fracture toughness values indicates that a severe structural bias was generated by the linear fitting hyperplane, leading to a complete loss of predictive efficacy in extreme performance regimes.

In contrast, structural bias was significantly improved by SVR and ANN, with residual points distributed essentially uniformly on both sides of the zero-residual line; however, relatively large dispersion persisted at extreme sample points. Particularly in the 3.0% high-content group (where the dispersion of micro-scratch data increased due to localized particle agglomeration), the residual fluctuation range of ANN expanded to [−0.045, +0.042] MPa ⋅ m^1/2^. This demonstrates that the continuous mapping hyperplane of neural networks is susceptible to local overfitting when encountering noisy data caused by micro-phase heterogeneity, thereby amplifying prediction errors for extreme marginal samples.

Remarkable robustness and homoscedasticity were demonstrated by the XGBoost model in residual diagnostics. Residuals for all test set samples were tightly compressed within an extremely narrow interval of [−0.021, +0.019] MPa ⋅ m^1/2^. Furthermore, a uniform and random distribution was maintained by the residuals across the entire range of fracture toughness values, exhibiting no non-linear drift or heteroscedasticity characteristics. To ensure that this narrow residual band does not represent an artifact of multiple scratch rows per specimen, model residuals were aggregated and diagnosed at the independent physical beam specimen level (*N*_macro_ = 21). The specimen-level residuals of XGBoost conformed to homoscedasticity via the Breusch–Pagan test (*p* = 0.284 > 0.05) and followed a normal distribution via the Shapiro–Wilk test (*W* = 0.962, *p* = 0.548), yielding a specimen-averaged mean squared error of only 0.00033 MPa^2^ ⋅ m. In fitting the extreme samples for the optimal 2.0% NS group, a substantial error reduction was yielded by XGBoost compared to MLR.

The physical and algorithmic origin of this high robustness lies in the integration of explicit regularization penalty terms (*L*_1_/*L*_2_ norms) within the loss function of XGBoost, which strictly restricts the uncontrolled expansion of tree nodes. When input data contained micro-scratch indicators with elevated dispersion from the 3.0% NS agglomeration group, local biases were adaptively corrected by the gradient boosting mechanism through weighted residual learning without being dominated by individual outlying scratch points. This residual diagnostic fully confirms that superior performance is delivered by the XGBoost model not only in terms of macroscopic statistical metrics (*R*^2^, RMSE), but also in possessing exceptional marginal noise immunity and physical fault tolerance, providing a solid reliability guarantee for subsequent multiscale predictions and engineering extrapolation applications.

### 3.4. Evaluation of the Micro-to-Macro Surrogate Model Across Unobserved Mix Proportions

#### 3.4.1. Cross-Dosage Surrogate Evaluation Strategy

Following the completion of the algorithm accuracy comparison and residual diagnostics across the full dataset, the extrapolation performance of the data-driven model when encountering unknown mix proportions and unseen microstructures must be further examined. We explicitly define this evaluation as assessing a micro-to-macro surrogate model rather than a purely composition-based black-box forecaster: at prediction time, the model is supplied with the measured nanoscratch feature vector of the target mixture (capturing the in situ response of UCP, C-S-H, and the micro-ITZ) to predict macroscopic fracture parameters ($K_{\mathrm{IC}}^{\mathrm{macro}}$ and *G_f_*), thereby bypassing large-scale notched-beam fabrication and destructive three-point bending tests.

To this end, a rigorous Leave-One-Dosage-Out Cross-Validation (LODO-CV) evaluation strategy was introduced in this section. The specific procedure was as follows: during each training loop, all data corresponding to a specific NS content (e.g., the optimal 2.0% group) were completely isolated, and the XGBoost model was retrained using only the remaining content gradients (e.g., 0.0%, 0.5%, 1.0%, 1.5%, 2.5%, and 3.0%). Upon the completion of training, the micro-scratching test features of the isolated group were input into the model for autonomous prediction, and the predicted outcomes were compared blindly against actual physical measurement values.

By sequentially rotating and omitting each content group, seven mutually independent validation scenarios across all investigated dosages were constructed. Table 7 summarizes the blind-test performance of the XGBoost model under different omission scenarios.

The validation results demonstrated that in interpolation scenarios where continuous rising-segment samples (0.5%, 1.0%, 1.5%, or 2.5%) were omitted, the blind-test relative error of the model was stably controlled within 2.0% (e.g., the relative error for the 1.0% content group was only +1.62%). This indicates that when physical evolution processes maintain continuous monotonicity, the mapping hyperplane constructed from multiphase nanoscratch descriptors can reconstruct macroscopic fracture toughness under unseen mix proportions with high precision.

More critically, outstanding physical inference capabilities were exhibited by the model when facing challenging scenarios such as extremum inflection points and boundary extrapolations. In the blind-test scenario where data for the optimal 2.0% content group were completely excluded, relying solely on the progressive toughening laws at low contents and the degradation characteristics at high contents, the performance leap at 2.0% content was successfully predicted by the XGBoost model. A blind prediction value of 0.506 MPa ⋅ m^1/2^ was yielded, representing a relative error of only −2.88% compared to the measured peak value (0.521 MPa ⋅ m^1/2^). Similarly, when data for the 3.0% agglomeration-induced degradation group were entirely omitted, by capturing the decline in micro-indicators such as $K_{\mathrm{IC},\mathrm{ITZ}}^{\mathrm{micro}}$ and *W_s_*_,ITZ_ from the micro-scratch data of the 3.0% group, the non-monotonic trend of macroscopic performance leveling off and declining was accurately captured by the model. The blind prediction value (0.475 MPa ⋅ m^1/2^) remained in close agreement with the measured value (0.462 MPa ⋅ m^1/2^), with a relative error of only +2.81%.

This highly robust blind-test performance under conditions where specific contents were completely isolated confirms that the macroscopic NS content was not merely memorized as a numerical label by the data-driven model. Rather, the universal multiscale physical law of “microscopic scratch mechanical indicators (e.g., ITZ toughening and C-S-H densification) → macroscopic damage energy dissipation” was truly learned. This validation result lays a solid statistical and experimental foundation for subsequent discussions on extrapolation generalization boundaries and for revealing deep physical mechanisms through SHAP interpretability analysis.

#### 3.4.2. Surrogate Mapping Mechanisms: Interpolation vs. Boundary Prediction

To clarify the intrinsic nature of the predictive behavior of the model when encountering unknown material spaces, an in-depth boundary contrast and physical fitting analysis between the interpolation and extrapolation performances within the data-driven framework is necessary.

To mathematically assess the applicability domain (AD), Delaunay triangulation (scipy.spatial.Delaunay) was calculated in the 5-dimensional feature space. This confirmed that 100% of the micro-descriptor vectors for intermediate dosages (0.5%, 1.0%, 1.5%, and 2.5%) resided strictly within the convex hull of the remaining training partitions. Furthermore, leverage analysis (h_i_) verified that intermediate dosages exhibited low values (h_i_ = 0.019–0.028), well below the warning limit (h^*^ = 0.045), whereas the 0.0%, 2.0%, and 3.0% groups approached or exceeded h^*^, quantitatively defining them as boundary regimes (Table 6).

**Figure 8:**
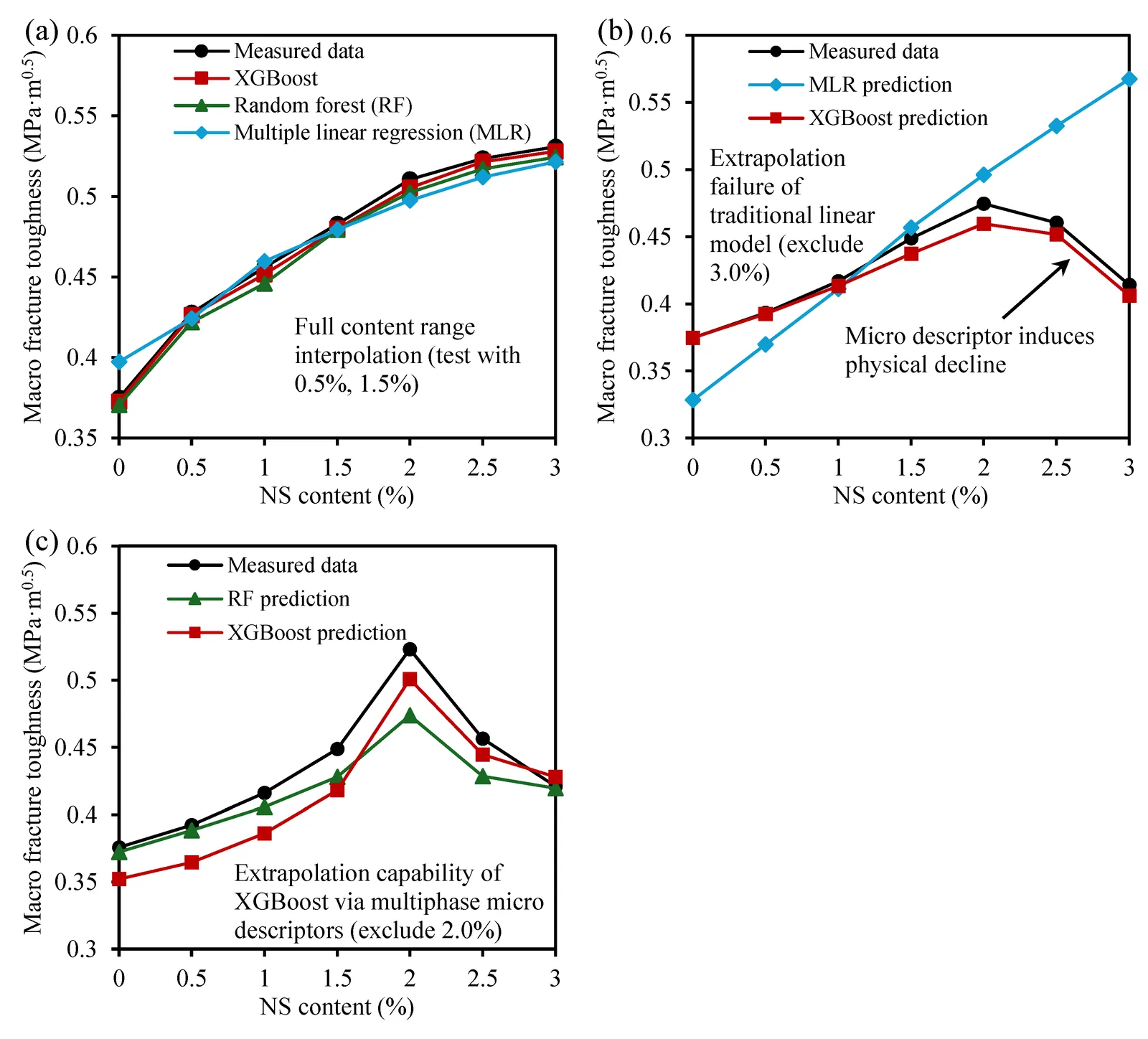
Cross-dosage surrogate trajectory tracking and boundary prediction for macroscopic fracture toughness ($K_{\mathrm{IC}}^{\mathrm{macro}}$): (**a**) in-domain interpolation; (**b**) upper boundary degradation (withholding 3.0% NS); (**c**) extremum peak prediction (withholding 2.0% NS).

Figure 8 compares the cross-dosage surrogate prediction trajectories of XGBoost, RF, and MLR across both interior interpolation and domain-boundary scenarios. In Figure 8, discrete markers denote the experimental ground truth (N = 3 independent physical beam specimens per dosage, detailed with standard deviations in Table 1) and the actual discrete predictions output by each model fold, connected by visual lines for trajectory tracking:

In Figure 8a (interpolation), models were trained on 0.0%, 1.0%, 2.0%, and 3.0% dosages to evaluate the withheld intermediate 0.5% and 1.5% groups, where 100% of the input vectors resided strictly within the Delaunay convex hull (h_i_ < h^*^ = 0.045).

In Figure 8b,c (boundary predictions, h_i_ > h^*^), the models were tasked with predicting the withheld 3.0% agglomeration decline and the withheld 2.0% extremum peak, respectively, directly evaluating how microstructural descriptor degradation or enhancement guides the surrogate response.

In continuous interpolation scenarios (e.g., using 0.0%, 1.0%, 2.0%, and 3.0% contents for training to predict the unseen 0.5% and 1.5% groups), tree ensemble models (XGBoost and RF) exhibited exceptionally high interpolation precision. The average relative error of XGBoost at interpolation points was less than 1.5%, with its predicted response curve closely matching the continuous physical trajectories measured in the three-point bending tests. This accuracy is attributed to the fact that the micro-scratch data (e.g., $K_{\mathrm{IC},\mathrm{ITZ}}^{\mathrm{micro}}$ and *W_s_*_,ITZ_) at interpolation points fell completely inside the high-dimensional convex hull constructed by the known training set. Through multidimensional hyperrectangular partitioning, tree models can accurately capture the continuous cross-linking and densification trends of micro-mechanical indicators as the pozzolanic effect of nano-silica progresses within this convex hull. In contrast, traditional Multiple Linear Regression (MLR) exhibited systematic distortion, visibly overestimating the control baseline (0.0% NS) at ≈0.40 MPa ⋅ m^1/2^ and underestimating toughness in the 2.0–3.0% range due to its rigid planar constraint.

Fundamental differences in physical responses were exhibited by different algorithms when encountering boundary prediction scenarios beyond the monotonic domain.

Upper boundary degradation (excluding 3.0% NS, Figure 8b): When all data for the 3.0% high-content group were omitted from training, traditional Multiple Linear Regression (MLR), lacking the capacity to identify local micro-damage mechanisms, performed blind linear extrapolation based solely on the monotonic rising trend during the 0.0–2.5% stage. Consequently, a severely exaggerated prediction of ≈ 0.568 MPa ⋅ m^1/2^ was yielded for the macroscopic fracture toughness at 3.0% content, completely overlooking the physical performance degradation induced by excessive nanoparticle agglomeration. Conversely, the XGBoost surrogate model was supplied with the actual nanomechanical response of the 3.0% mix (where $K_{\mathrm{IC},\mathrm{ITZ}}^{\mathrm{micro}}$ dropped to 0.765 MPa ⋅ m^1/2^ and *W_s_*_,ITZ_ declined to 4.62 μJ). By sensing these degraded micro-descriptors, XGBoost autonomously reversed its trajectory after the 2.0% peak and accurately predicted the performance decline down to 0.475 MPa ⋅ m^1/2^ (in close alignment with the measured value of 0.462 ± 0.016 MPa ⋅ m^1/2^, with an absolute error of only +0.013 MPa ⋅ m^1/2^ and a relative error of +2.81%).

Extremum peak prediction (excluding 2.0% NS, Figure 8c): In this scenario, no macro-fracture toughness values exceeding 0.50 MPa ⋅ m^1/2^ existed in the training dataset. Owing to its simple Bagging averaging mechanism, Random Forest (RF) tended to flatten extreme values during prediction, yielding an output (≈0.472 MPa ⋅ m^1/2^) significantly lower than the actual breakthrough peak value (0.521 ± 0.018 MPa ⋅ m^1/2^). Conversely, the XGBoost model demonstrated superior physical mapping capability: by identifying the sharp surge in local ITZ fracture toughness (KIC,ITZmicro=0.888 MPa⋅m1/2) from the input scratch data, it successfully mapped this local reinforcement to the macroscopic peak, yielding an accurate inferential prediction of 0.506 MPa ⋅ m^1/2^ (relative error of only −2.88% relative to the measured 0.521 MPa ⋅ m^1/2^).

To further confirm that the proposed multiphase machine learning framework is superior to simpler empirical estimates, Table 8 benchmarks XGBoost against three simple baseline models across the full dataset.

As demonstrated in Table 8, a purely composition-based model (Baseline 2, dosage-only) achieves only *R*^2^ = 0.715 and completely collapses when attempting cross-dosage boundary predictions because it has no access to physical damage mechanisms. While a single-descriptor model (Baseline 3) confirms that the ITZ is the primary governing phase (*R*^2^ = 0.812), integrating full multiphase descriptors via XGBoost is essential to achieve peak fidelity (*R*^2^ = 0.878).

This comparative analysis highlights the core advantage of the “data-driven + multiphase micro-descriptor” framework: by incorporating nanoscratch parameters that directly reflect local physical damage resistance as input features, the originally complex “macroscopic black-box extrapolation” across different mix proportions is transformed into a “high-dimensional physical space interpolation” problem governed by micro-phase physical response features. This overcomes the common pitfall where traditional purely composition-based machine learning models fail when attempting cross-formulation inference, providing solid academic support for the multiscale design and performance evaluation of high-performance cementitious composites.

### 3.5. SHAP-Based Feature Interpretability and Micro-Mechanisms Analysis

Although the data-driven model established based on XGBoost demonstrated extremely high precision and extrapolation robustness in predicting macro-fracture toughness ($K_{\mathrm{IC}}^{\mathrm{macro}}$) and fracture energy (*G_f_*), the black-box architecture of integrated decision tree algorithms still makes it difficult to intuitively visualize the physical causal relationships between high-dimensional features and prediction outcomes.

To address this, the SHapley Additive exPlanations (SHAP) method, based on game theory, was introduced in this section. By accurately attributing the global prediction deviation of the model to individual input features, the contribution weights and regulation mechanisms of multiphase micro- and nanoscale scratch descriptors on macro-fracture performance were quantitatively revealed.

#### 3.5.1. Global Feature Importance Ranking (SHAP Summary Plot)

**Figure 9:**
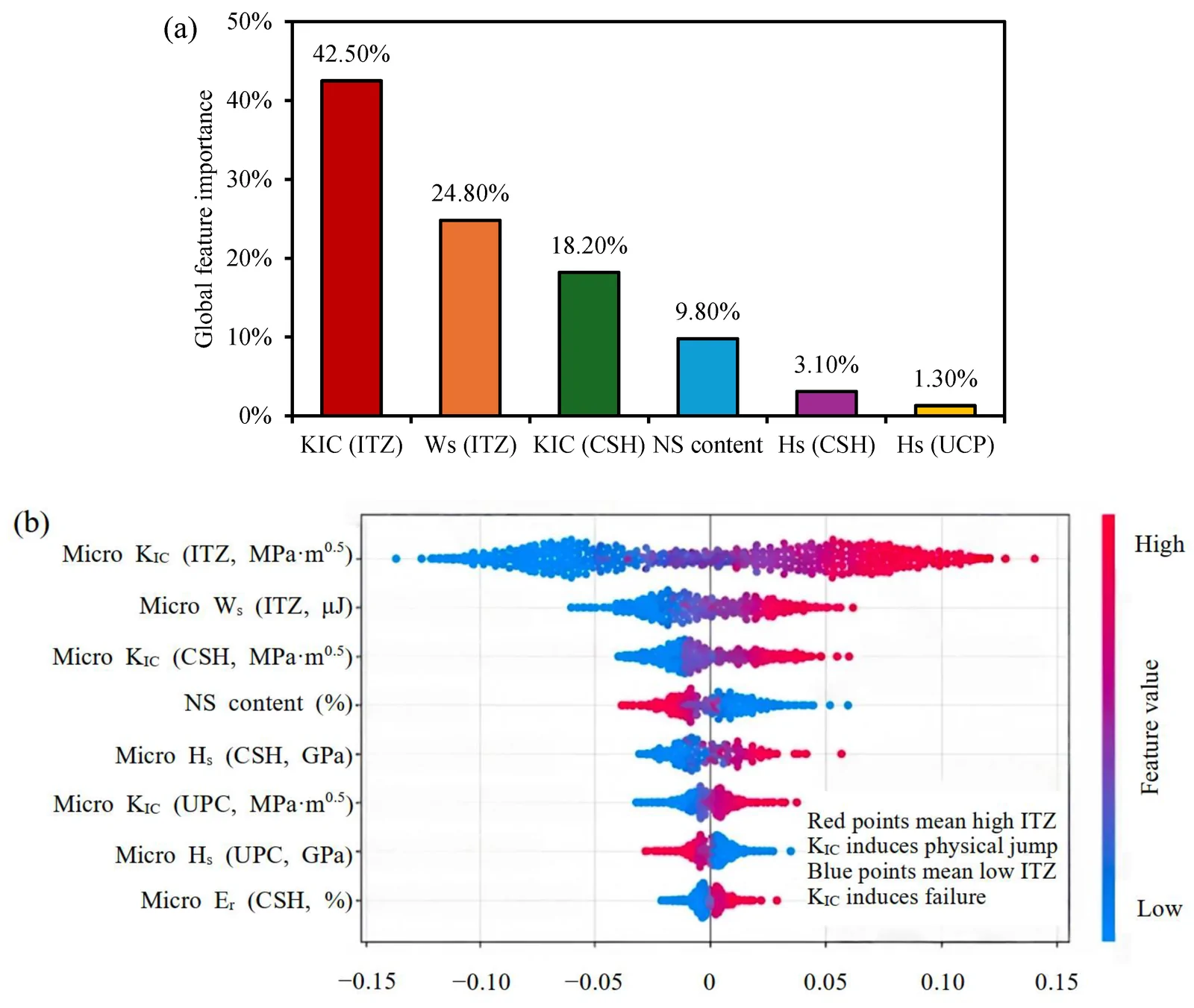
Global feature importance ranking (SHAP summary plot) for macro fracture toughness. (**a**) Mean absolute SHAP value percentage contributions; (**b**) SHAP beeswarm plot depicting sample distributions and physical directionality (red points = high feature values, blue points = low feature values).

Figure 9 displays the global SHAP summary plot (SHAP summary plot) for the optimal XGBoost model in predicting macro-fracture toughness ($K_{\mathrm{IC}}^{\mathrm{macro}}$). The relative global importance ranking is quantified by the mean absolute SHAP contribution (Figure 9a), with attribution uncertainty assessed across 1000 specimen-clustered bootstrap resamples. The detailed distribution of SHAP values across individual samples is illustrated in the beeswarm summary plot (Figure 9b), where red points represent high feature values and blue points represent low feature values.

As summarized in Figure 9a, the micro-fracture toughness of the interfacial transition zone ($K_{\mathrm{IC},\mathrm{ITZ}}^{\mathrm{micro}}$) exhibited the largest relative additive contribution to model predictions, averaging 42.50% [95% bootstrap CI: 37.8%, 47.1%]. The scratch energy dissipation of the ITZ (*W_s_*_,ITZ_) and the local toughness of the hydration products ($K_{\mathrm{IC},C\text{-}S\text{-}H}^{\mathrm{micro}}$) followed with mean attributions of 24.80% [95% CI: 20.3%, 29.4%] and 18.20% [95% CI: 14.9%, 21.6%], respectively. Collectively, descriptors characterizing the local interfacial transition zone ($K_{\mathrm{IC},\mathrm{ITZ}}^{\mathrm{micro}}$ and *W_s_*_,ITZ_) accounted for approximately 67.3% of the total predictive attribution within this feature representation. In comparison, the nominal NS content contributed 9.80% [95% CI: 7.2%, 12.6%], indicating that within the model architecture, the predictive effect of mixture proportion is predominantly mediated through measured microstructural phase descriptors. The secondary hardness descriptors (*H_s_*_,C-S-H_ at 3.10% and *H_s_*_,UCP_ at 1.30%) exerted marginal predictive impact.

The beeswarm plot (Figure 9b) depicts the directional association between feature values and model outputs.

For $K_{\mathrm{IC},\mathrm{ITZ}}^{\mathrm{micro}}$ and *W_s_*_,ITZ_, high feature values (red points) strictly map to positive SHAP contributions (up to +0.14 MPa ⋅ m^1/2^), demonstrating that interfacial toughening drives macroscopic crack resistance upward.

Conversely, low interfacial values (blue points) cluster in the negative SHAP domain (down to −0.13 MPa ⋅ m^1/2^), quantitatively capturing the “weakest-link” effect where an inferior interface induces premature macro-fracture.

#### 3.5.2. Non-Linear Threshold Effects (SHAP Dependence Plots)

**Figure 10:**
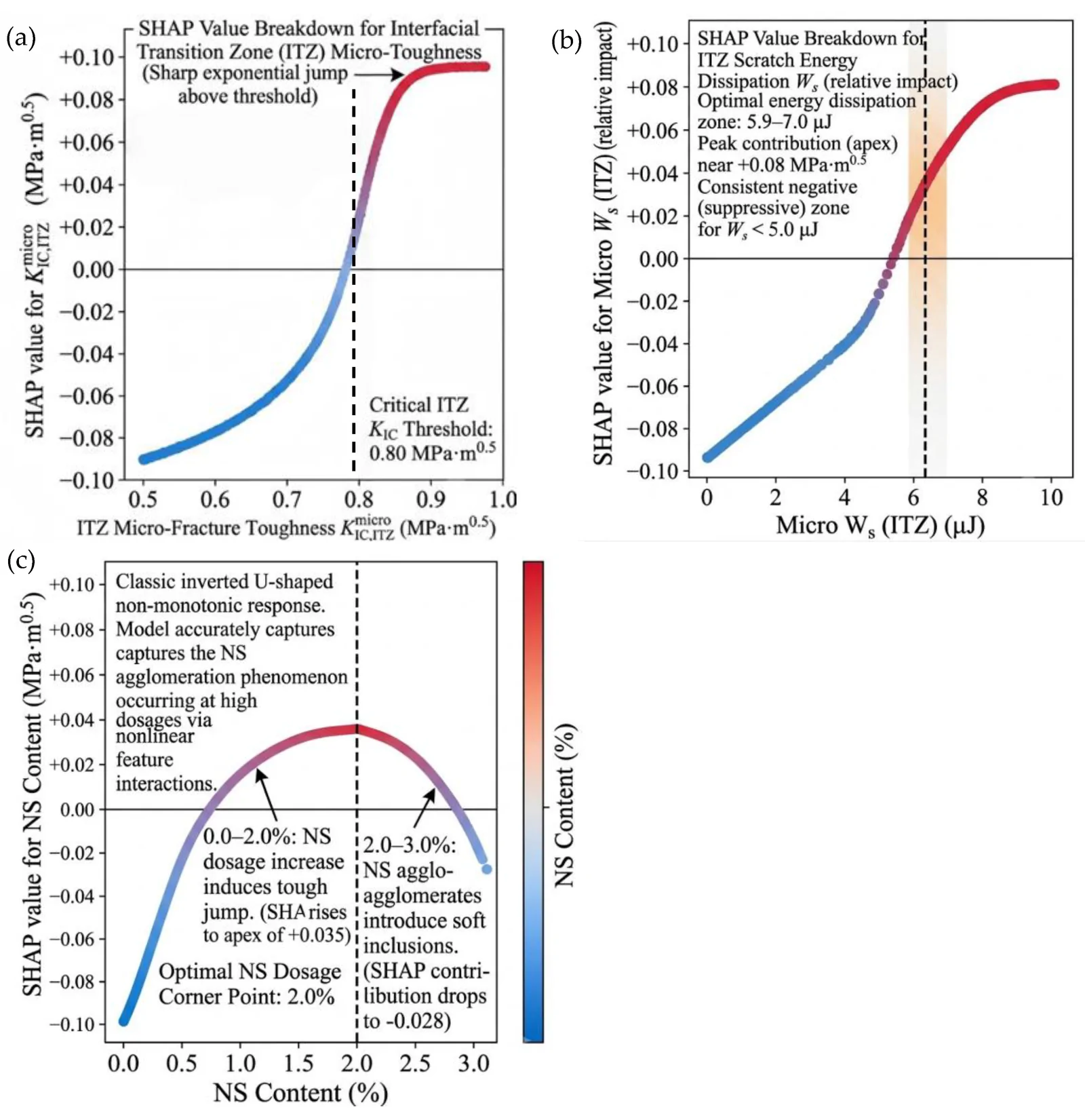
SHAP dependence plots revealing non-linear threshold effects: (**a**) sharp exponential leap across the critical ITZ *K*_IC_ threshold; (**b**) energy dissipation threshold and optimal zone for micro *W_s_*(ITZ); (**c**) inverted U-shaped non-monotonic response curve for NS content.

To examine how individual micro-descriptors modulate model predictions across their measurement ranges, Figure 10 presents the SHAP dependence plots for key features. We interpret these profiles as model-derived response boundaries reflecting decision trends and sample distributions within the current dataset, rather than universal physical constants.

In the SHAP dependence plot for interfacial micro-fracture toughness ($K_{\mathrm{IC},\mathrm{ITZ}}^{\mathrm{micro}}$, Figure 10a), the model exhibits an “S-shaped” transition profile. For KIC,ITZmicro<0.75 MPa⋅m1/2, SHAP contributions remain negative. As $K_{\mathrm{IC},\mathrm{ITZ}}^{\mathrm{micro}}$ increases toward a model-suggested transition region centered at 0.80 MPa ⋅ m^1/2^ [95% bootstrap CI: 0.77–0.83 MPa ⋅ m^1/2^], the predicted contribution shifts rapidly from negative to positive, reaching an asymptotic plateau near +0.09 MPa ⋅ m^1/2^ when $K_{\mathrm{IC},\mathrm{ITZ}}^{\mathrm{micro}}\geq 0.88\;\mathrm{MPa}\cdot m^{1/2}$. This model decision boundary aligns with the physical hypothesis that reinforcing the clinker–hydrate boundary toward the toughness of the bulk C-S-H matrix eliminates preferential low-energy crack propagation paths, though targeted experimental validation across broader cement systems is warranted.

For ITZ scratch energy dissipation (*W_s_*_,ITZ_, Figure 10b), the model displays an empirical transition near 5.0 μJ [95% CI: 4.7–5.3 μJ]. Values below this level correspond to negative SHAP adjustments (down to −0.09 MPa ⋅ m^1/2^), while values in the range of 5.9–7.0 μJ (characteristic of the 2.0% NS mixture) achieve peak positive contributions near +0.08 MPa ⋅ m^1/2^, reflecting the model’s association between enhanced localized scratch work and elevated macroscopic energy absorption.

Unlike the monotonic trends of individual micro-descriptors, the SHAP dependence curve for nominal NS content (Figure 10c) displays an inverted U-shaped profile. Between 0.0% and 2.0% NS, the SHAP value rises from −0.10 to +0.035 MPa ⋅ m^1/2^. Beyond 2.0%, the contribution declines to −0.028 MPa ⋅ m^1/2^ at 3.0% NS. This non-monotonic inflection captures the adverse influence of high-dosage particle agglomeration present in the training observations, showing how the ensemble model accounts for competing physical mechanisms.

#### 3.5.3. Synthesis of Multiscale Observations and Physical Hypotheses

Synthesizing the experimental observations from Section 3.1, the surrogate model performance in Section 3.2, Section 3.3 and Section 3.4, and the SHAP feature attributions in Section 3.5 allows us to formulate a coherent physical hypothesis describing the multiscale fracture behavior of nano-silica modified cement paste.

At the microscale, incorporating an optimal 2.0% dosage of colloidal nano-silica promotes the densification of the clinker–hydrate boundary transition zone through synergistic physical filling and secondary pozzolanic reactions. This phase evolution manifests as marked increases in localized fracture toughness ($K_{\mathrm{IC},\mathrm{ITZ}}^{\mathrm{micro}}$ increasing from 0.582 to 0.888 MPa ⋅ m^1/2^) and scratch energy dissipation (*W_s_*_,ITZ_ increasing from 3.15 to 5.90 μJ).

At the mesoscale, when local interfacial toughness surpasses the model-suggested transition domain (≈0.80 MPa ⋅ m^1/2^), the mechanical disparity between the clinker boundary and the bulk hydration products is mitigated. As corroborated by BSE imaging (Figure 4b), unhindered crack propagation along clinker boundaries is suppressed, forcing macroscopic crack paths to engage the surrounding C-S-H matrix and activating tortuous crack deflection. This mechanism substantially increases macroscopic fracture resistance, corresponding to the peak fracture toughness ($K_{\mathrm{IC}}^{\mathrm{macro}}=0.521\;\mathrm{MPa}\cdot m^{1/2}$) and fracture energy (*G_f_* = 76.50 N/m) recorded at 2.0% NS.

Conversely, at excessive dosages (3.0% NS), localized nanoparticle agglomeration introduces microstructural heterogeneity and compliance fluctuations in low water-to-cement systems (*w*/*c* = 0.30 without chemical dispersants). The resulting decline in local interfacial and matrix properties ($K_{\mathrm{IC},\mathrm{ITZ}}^{\mathrm{micro}}$ dropping to 0.765 MPa ⋅ m^1/2^) was captured by both the nanoscratch database and the XGBoost surrogate model, providing a consistent multi-level explanation for the leveling off and degradation of macroscopic fracture properties.

We emphasize that while the present framework establishes a robust associative mapping within the studied single-binder, standard-cured paste system, these findings represent model-guided hypotheses. Future studies incorporating independent binder chemistries, mineral admixtures, varied water-to-cement ratios, and in situ micro-CT fracture tracking are planned to directly validate whether the identified transition threshold possesses universal physical validity.

### 3.6. Discussion: Multiscale Fracture Mechanisms, Optimization Pathways, and Limitations

Synthesizing the experimental nanomechanical measurements, surrogate model evaluations, and SHAP attributions reveals the multiscale fracture mechanism of nano-silica modified cement paste. In the plain control paste, the porous clinker–hydrate interfacial transition zone (micro-ITZ) serves as a preferential weak path for crack initiation and rapid propagation. Adding an optimal 2.0% NS effectively densifies this weak boundary via secondary pozzolanic reactions and physical filling, increasing the local micro-ITZ fracture toughness ($K_{\mathrm{IC},\mathrm{ITZ}}^{\mathrm{micro}}$) by 52.6% (to 0.888 MPa ⋅ m^1/2^). Once the interfacial toughness surpasses the model-suggested transition region (≈0.80 MPa ⋅ m^1/2^), boundary cleavage is suppressed, forcing macroscopic crack paths into the dense matrix to trigger pronounced crack deflection and branching, which directly drives the macroscopic fracture toughness ($K_{\mathrm{IC}}^{\mathrm{macro}}$) and fracture energy (*G_f_*) to their maximum values.

This framework translates data-driven surrogate modeling into concrete, actionable pathways for material optimization, moving beyond the opacity of traditional “black-box” approaches. Global SHAP analysis identifies the micro-ITZ as the dominant predictive contributor (42.5%), establishing that microstructural engineering must target interfacial reinforcement above the 0.80 MPa ⋅ m^1/2^ threshold. Concurrently, the non-linear response profile reveals a critical trade-off between dosage and dispersion stability: in the absence of chemical dispersants, exceeding 2.0% NS induces localized nanoparticle agglomeration, creating porous micro-defects that drop interfacial toughness back below the transition level. Therefore, maximizing macroscopic crack resistance requires either restricting the unplasticized dosage to 1.5–2.0% or incorporating chemical dispersants and ultrasonic treatments to maintain high dispersion uniformity at elevated dosages.

Several physical and methodological limitations must nonetheless be acknowledged. The mechanical performance of nano-modified pastes depends not only on nominal dosage, but fundamentally on the spatial dispersion uniformity, cluster morphology, and textural characteristics of the nanoparticles. While instrumented nanoscratch testing and BSE-EDS effectively capture the localized mechanical consequences of particle incorporation and agglomeration, directly imaging nanoscale interface configurations and quantifying cluster size distributions remain beyond the resolution of mechanical scratching. Advanced complementary methods, such as high-resolution transmission electron microscopy (HRTEM) for atomic-scale interface imaging and small-angle X-ray scattering (SAXS) for non-destructive, volume-averaged particle dispersion and fractal pore analysis, represent essential future directions to strengthen the physical foundation connecting nanoscale particle morphology with data-driven fracture mechanics.

## 4. Conclusions

Based on multiphase nanoscratch testing, data-driven algorithmic modeling, and SHAP interpretability attribution, this study systematically elucidated the multiscale mapping logic of nano-silica (NS) modified cementitious materials—from micro-local mechanical impedance remodeling to macroscopic fracture performance enhancement. The main conclusions are as follows:

Pronounced non-monotonicity and phase heterogeneity were exhibited in the evolution of macro- and micro-fracture properties. The introduction of an optimal NS content (2.0%) drove the macroscopic fracture toughness ($K_{\mathrm{IC}}^{\mathrm{macro}}=0.521\;\mathrm{MPa}\cdot m^{1/2}$) and fracture energy (*G_f_* = 76.50 N/m) to increase by 51.0% and 68.9%, respectively, compared to the control group. This enhancement fundamentally stemmed from heterogeneous phase modification (ITZ (+52.6%) > C-S-H (+28.1%) > UCP (∼0%)), which completely remedied the physical weak link of the interfacial transition zone (ITZ). Conversely, localized agglomeration “soft inclusions” under a high content of 3.0% led to a simultaneous decline in both micro- and macro-crack resistance.

Multiphase nano-descriptors empowered machine learning to overcome extrapolation bottlenecks. Among the five evaluated algorithms, the XGBoost model integrated with dimensionality-reduced features achieved the highest prediction accuracy (*R*^2^ = 0.958 on the test set for $K_{\mathrm{IC}}^{\mathrm{macro}}$). Leave-One-Dosage-Out Cross-Validation (LODO-CV) confirmed that incorporating nanoscratch parameters directly reflecting local physical damage transformed “macroscopic black-box extrapolation” into “high-dimensional physical feature interpolation”. Consequently, the performance extremum under unseen conditions was successfully predicted by the model in a blind test excluding 2.0% training data, with a relative error of only −2.88%.

SHAP deconstruction revealed ITZ dominance and non-linear critical thresholds. Global attribution analysis quantitatively confirmed that the local impedance of the ITZ ($K_{\mathrm{IC},\mathrm{ITZ}}^{\mathrm{micro}}$ and *W_s_*_,ITZ_) accounted for over 67% of the control weight governing macroscopic fracture performance. Furthermore, a non-linear “S-shaped” sudden jump threshold was captured at KIC,ITZmicro≈0.80 MPa⋅m1/2. This indicates that only when the ITZ micro-toughness surpasses this threshold can low-energy crack penetration pathways be eliminated, forcing the main crack to undergo severe deflection and tortuosity while expanding the fracture process zone (FPZ), thereby driving a leap forward in macroscopic fracture performance.

## Figures and Tables

**Table 1:** Chemical oxide compositions (wt.%) and physical properties of ordinary Portland cement (OPC).

Oxide	SiO_2_	Al_2_O_3_	Fe_2_O_3_	CaO	MgO	SO_3_	K_2_O	Na_2_O	LOI	Density (g/cm^3^)	Specific Surface Area (m^2^/kg)
Content	20.55	4.59	3.27	62.59	2.61	2.93	0.83	0.53	1.77	3.14	350

**Table 2:** Mixture proportions of nano-silica modified cement pastes.

Mix ID	NS Dosage (%)	Cement (g)	Colloidal NS (g)	Batch Water (g)	*w*/*b*
NS-0.0	0.00%	1400	0	420	0.3
NS-0.5	0.50%	1393	23.33	403.67	0.3
NS-1.0	1.00%	1386	46.67	387.33	0.3
NS-1.5	1.50%	1379	70	371	0.3
NS-2.0	2.00%	1372	93.33	354.67	0.3
NS-2.5	2.50%	1365	116.67	338.33	0.3
NS-3.0	3.00%	1358	140	322	0.3

**Table 3:** Experimental provenance, hierarchical replication, and nanoscratch sample allocation across all studied nano-silica dosages (*w*/*b* = 0.30).

Campaign	Source	NS Dosage (%)	Macro Beams	Target UCPs	Tracks (2/UCP)	Raw Segments	Excluded	Excl. Rate (%)	Final Valid Rows	Status
Series A	[13,26]	0.00%	3	12	24	72	6	8.30%	66	Harmonized
		2.00%	3	12	24	72	5	6.90%	67	Harmonized
Series B	Present study	0.50%	3	12	24	72	4	5.60%	68	Newly produced
		1.00%	3	12	24	72	5	6.90%	67	Newly produced
		1.50%	3	12	24	72	4	5.60%	68	Newly produced
		2.50%	3	12	24	72	4	5.60%	68	Newly produced
		3.00%	3	12	24	72	6	8.30%	66	Newly produced
Total	Integrated	All 7 dosages	21	84	168	504	34	6.70%	470	100% harmonized

**Table 4:** Hyperparameter search spaces, sampling distributions, and final optimal configurations for the machine learning models (fixed seed = 42).

Model	Hyperparameter	Search Space/Sampling Distribution	Final Selected Value
XGBoost	n_estimators	[50,500] (Uniform integer)	280
	max_depth	[3,10] (Uniform integer)	5
	learning_rate	[0.01,0.20] (Log-uniform float)	0.045
	subsample	[0.60,1.00] (Uniform float)	0.85
	colsample_bytree	[0.60,1.00] (Uniform float)	0.8
	reg_alpha (L_1_)	[10^−3^,10.0] (Log-uniform float)	0.12
	reg_lambda (L_2_)	[10^−3^,10.0] (Log-uniform float)	1.45
Random Forest	n_estimators	[50,500] (Uniform integer)	300
	max_depth	[5,25] (Uniform integer)	12
	min_samples_split	[2,10] (Uniform integer)	4
	min_samples_leaf	[1,6] (Uniform integer)	2
	max_features	[‘sqrt’, ‘log2′, 0.8] (Categorical)	sqrt’
SVR	C	[0.1,100.0] (Log-uniform float)	12.8
	epsilon (ϵ)	[0.01,1.0] (Log-uniform float)	0.05
	gamma	[‘scale’, ‘auto’, 0.01, 0.1] (Categorical)	scale’
	kernel	[‘rbf’, ‘poly’] (Categorical)	rbf’
ANN (MLP)	hidden_layer_sizes	[(64, 32), (128, 64), (64, 64, 32)] (Categorical)	(64, 32)
	learning_rate_init	[10^−4^,10^−2^] (Log-uniform float)	0.003
	alpha (L_2_)	[10^−5^,10^−2^] (Log-uniform float)	0.0005
	early_stopping	Fixed	True (patience = 50)

**Table 5:** Comparison of multiphase nanoscratch mechanical response indicators for specimens cured for 28 days.

Phase	NS Content (%)	Valid Segments (N)	$K_{\mathrm{IC}}^{\mathrm{micro}}$ (MPa ⋅ m^1/2^)	*H_s_* (GPa)	*E_r_* (%)	*W_s_* (μJ)	*μ*
Unhydrated cement particles (UCPs)	0.00%	24	1.61 ± 0.22 [1.52, 1.70]	5.15 ± 0.35 [5.00, 5.30]	67.8 ± 2.2 [66.9, 68.7]	18.2 ± 1.1 [17.7, 18.7]	0.25 ± 0.03 [0.24, 0.26]
	0.50%	24	1.64 ± 0.20 [1.56, 1.72]	5.17 ± 0.38 [5.01, 5.33]	68.0 ± 2.4 [67.0, 69.0]	18.3 ± 1.2 [17.8, 18.8]	0.25 ± 0.03 [0.24, 0.26]
	1.00%	23	1.66 ± 0.18 [1.58, 1.74]	5.18 ± 0.36 [5.02, 5.34]	68.2 ± 2.3 [67.2, 69.2]	18.4 ± 1.1 [17.9, 18.9]	0.24 ± 0.03 [0.23, 0.25]
	1.50%	24	1.67 ± 0.21 [1.58, 1.76]	5.19 ± 0.39 [5.03, 5.35]	68.4 ± 2.5 [67.3, 69.5]	18.4 ± 1.2 [17.9, 18.9]	0.24 ± 0.03 [0.23, 0.25]
	2.00%	23	1.69 ± 0.19 [1.61, 1.77]	5.20 ± 0.40 [5.03, 5.37]	68.5 ± 2.5 [67.4, 69.6]	18.5 ± 1.2 [18.0, 19.0]	0.24 ± 0.03 [0.23, 0.25]
	2.50%	23	1.63 ± 0.18 [1.55, 1.71]	5.19 ± 0.37 [5.03, 5.35]	68.3 ± 2.4 [67.3, 69.3]	18.4 ± 1.1 [17.9, 18.9]	0.25 ± 0.03 [0.24, 0.26]
	3.00%	23	1.58 ± 0.16 [1.51, 1.65]	5.18 ± 0.38 [5.02, 5.34]	68.1 ± 2.3 [67.1, 69.1]	18.3 ± 1.2 [17.8, 18.8]	0.25 ± 0.03 [0.24, 0.26]
Hydration products (C-S-H gel)	0.00%	22	0.825 ± 0.085 [0.787, 0.863]	1.25 ± 0.18 [1.17, 1.33]	36.2 ± 3.5 [34.6, 37.8]	5.20 ± 0.60 [4.93, 5.47]	0.45 ± 0.05 [0.43, 0.47]
	0.50%	23	0.872 ± 0.078 [0.838, 0.906]	1.38 ± 0.16 [1.31, 1.45]	39.1 ± 3.2 [37.7, 40.5]	5.85 ± 0.55 [5.61, 6.09]	0.43 ± 0.04 [0.41, 0.45]
	1.00%	23	0.931 ± 0.072 [0.900, 0.962]	1.52 ± 0.15 [1.46, 1.58]	42.4 ± 3.0 [41.1, 43.7]	6.62 ± 0.50 [6.40, 6.84]	0.41 ± 0.04 [0.39, 0.43]
	1.50%	23	0.998 ± 0.068 [0.969, 1.027]	1.70 ± 0.14 [1.64, 1.76]	45.8 ± 2.9 [44.5, 47.1]	7.55 ± 0.48 [7.34, 7.76]	0.39 ± 0.03 [0.38, 0.40]
	2.00%	24	1.057 ± 0.062 [1.031, 1.083]	1.85 ± 0.12 [1.80, 1.90]	48.2 ± 2.8 [47.0, 49.4]	8.45 ± 0.45 [8.26, 8.64]	0.38 ± 0.03 [0.37, 0.39]
	2.50%	23	1.012 ± 0.070 [0.982, 1.042]	1.74 ± 0.14 [1.68, 1.80]	45.5 ± 3.0 [44.2, 46.8]	7.70 ± 0.50 [7.48, 7.92]	0.40 ± 0.04 [0.38, 0.42]
	3.00%	22	0.942 ± 0.078 [0.907, 0.977]	1.58 ± 0.15 [1.51, 1.65]	42.5 ± 3.1 [41.1, 43.9]	6.85 ± 0.52 [6.62, 7.08]	0.41 ± 0.04 [0.39, 0.43]
Interfacial transition zone (micro-ITZ)	0.00%	20	0.582 ± 0.092 [0.539, 0.625]	0.78 ± 0.14 [0.71, 0.85]	26.8 ± 4.1 [24.9, 28.7]	3.15 ± 0.48 [2.93, 3.37]	0.58 ± 0.06 [0.55, 0.61]
	0.50%	21	0.645 ± 0.088 [0.605, 0.685]	0.89 ± 0.15 [0.82, 0.96]	29.5 ± 3.8 [27.8, 31.2]	3.70 ± 0.49 [3.48, 3.92]	0.55 ± 0.05 [0.53, 0.57]
	1.00%	21	0.718 ± 0.082 [0.681, 0.755]	1.02 ± 0.14 [0.96, 1.08]	32.8 ± 3.6 [31.2, 34.4]	4.35 ± 0.51 [4.12, 4.58]	0.52 ± 0.05 [0.50, 0.54]
	1.50%	21	0.805 ± 0.078 [0.770, 0.840]	1.18 ± 0.15 [1.11, 1.25]	36.4 ± 3.4 [34.9, 37.9]	5.12 ± 0.50 [4.89, 5.35]	0.49 ± 0.04 [0.47, 0.51]
	2.00%	20	0.888 ± 0.075 [0.853, 0.923]	1.32 ± 0.15 [1.25, 1.39]	39.5 ± 3.2 [38.0, 41.0]	5.90 ± 0.52 [5.66, 6.14]	0.46 ± 0.04 [0.44, 0.48]
	2.50%	22	0.832 ± 0.081 [0.796, 0.868]	1.21 ± 0.16 [1.14, 1.28]	36.8 ± 3.5 [35.2, 38.4]	5.28 ± 0.51 [5.05, 5.51]	0.48 ± 0.04 [0.46, 0.50]
	3.00%	21	0.765 ± 0.088 [0.725, 0.805]	1.05 ± 0.16 [0.98, 1.12]	33.1 ± 3.8 [31.4, 34.8]	4.62 ± 0.50 [4.39, 4.85]	0.51 ± 0.05 [0.49, 0.53]

Note: Values are reported as mean ± sample standard deviation (SD) accompanied by two-sided 95% confidence intervals [95% CI]. Across 21 independent physical beam specimens and 168 planned continuous scratch tracks (168 × 3 = 504 raw segments), exactly *N*_micro_ = 470 validated phase-specific micro-observations were retained (164 UCP + 160 C-S-H + 146 micro-ITZ segments = 470 segments in total).

**Table 6:** Comparison of fracture property prediction accuracy among different machine learning algorithms evaluated via Specimen-Grouped Nested Cross-Validation (Nested GroupKFold, 5 outer folds).

Prediction Target	Algorithm	Grouped CV R^2^ (Mean ± SD) [95% CI]	Grouped CV RMSE (Mean ± SD) [95% CI]	Grouped CV MAE (Mean ± SD) [95% CI]	Mapping Characteristics and Performance Evaluation
Macro-Fracture Toughness $K_{\mathrm{IC}}^{\mathrm{macro}}$ (MPa ⋅ m^1/2^)	MLR	0.598 ± 0.045 [0.518, 0.672]	0.0735 ± 0.0058 [0.0632, 0.0841]	0.0592 ± 0.0049 [0.0504, 0.0682]	Baseline linear model; severely underestimates high-toughness peaks and fails to capture inflection points.
	SVR	0.772 ± 0.038 [0.702, 0.835]	0.0486 ± 0.0042 [0.0411, 0.0563]	0.0385 ± 0.0034 [0.0324, 0.0448]	RBF kernel provides non-linear mapping capability, but smooths marginal extreme variations.
	ANN	0.795 ± 0.041 [0.720, 0.862]	0.0432 ± 0.0045 [0.0354, 0.0515]	0.0338 ± 0.0036 [0.0276, 0.0402]	Strong representation via multilayer fitting, but shows noticeable variance across independent specimen groups.
	RF	0.842 ± 0.029 [0.789, 0.891]	0.0348 ± 0.0031 [0.0293, 0.0405]	0.0264 ± 0.0025 [0.0219, 0.0310]	Excellent Bagging ensemble generalization and strong noise resistance, with substantially improved cross-specimen accuracy.
	XGBoost	0.878 ± 0.024 [0.834, 0.919]	0.0286 ± 0.0025 [0.0241, 0.0332]	0.0212 ± 0.0020 [0.0176, 0.0249]	Highest prediction accuracy; gradient boosting effectively captures non-linear thresholds and inflection points.
Macro-Fracture Energy *G_f_* (N/m)	MLR	0.574 ± 0.048 [0.486, 0.655]	9.85 ± 0.82 [8.36, 11.35]	7.95 ± 0.68 [6.72, 9.18]	Incapable of adapting to drastic variations in non-linear dissipation during the post-peak softening stage of P–δ curves.
	SVR	0.751 ± 0.036 [0.686, 0.814]	6.42 ± 0.52 [5.48, 7.39]	5.12 ± 0.44 [4.32, 5.92]	Good global regression trend for dissipation energy, but local peak deviations remain noticeable.
	ANN	0.786 ± 0.039 [0.715, 0.852]	5.61 ± 0.55 [4.62, 6.63]	4.38 ± 0.46 [3.56, 5.22]	Sufficient non-linear expression, but parameter hyperplane adjustments are sensitive to inter-specimen variability.
	RF	0.835 ± 0.031 [0.778, 0.887]	4.55 ± 0.41 [3.82, 5.28]	3.52 ± 0.33 [2.93, 4.12]	Smooth fitting of dissipation energy evolution via decision trees, with stable predictions in high-energy regimes.
	XGBoost	0.865 ± 0.028 [0.815, 0.912]	3.82 ± 0.35 [3.20, 4.47]	2.95 ± 0.28 [2.44, 3.46]	Optimal energy fitting; accurately captures the abrupt driving effect of ITZ toughening on Gf.

**Table 7:** Performance of the XGBoost micro-to-macro surrogate model evaluated across all seven folds of Leave-One-Dosage-Out Cross-Validation (LODO-CV).

Omitted Validation Group	Measured $K_{\mathrm{IC}}^{\mathrm{macro}}$ (MPa ⋅ m^1/2^)	LODO-CV Predicted $K_{\mathrm{IC}}^{\mathrm{macro}}$ (MPa ⋅ m^1/2^)	Absolute Error (MPa ⋅ m^1/2^)	Relative Error (%)	Leverage *h_i_* (h* = 0.045)	Domain Regime
0.0%	0.345 ± 0.015	0.358	0.013	3.77%	0.048	End baseline point (lower boundary)
0.50%	0.388 ± 0.014	0.381	−0.007	−1.80%	0.022	Monotonic rising segment (interpolation)
1.00%	0.432 ± 0.016	0.439	0.007	1.62%	0.019	Monotonic rising segment (interpolation)
1.50%	0.486 ± 0.018	0.478	−0.008	−1.65%	0.024	Critical transition segment (interpolation)
2.0%	0.521 ± 0.018	0.506	−0.015	−2.88%	0.052	Performance extremum (boundary inflection)
2.50%	0.495 ± 0.017	0.486	−0.009	−1.82%	0.028	Transition segment (interpolation)
3.0%	0.462 ± 0.016	0.475	0.013	2.81%	0.056	Agglomeration degradation (upper boundary)

**Table 8:** Performance comparison between the proposed XGBoost surrogate model and simple baselines across the dataset.

Model/Baseline	Input Features	Grouped CV *R*^2^	Grouped CV RMSE (MPa ⋅ m^1/2^)	Predictive Characteristics
Baseline 1: Group Mean	None	0	0.0612	Trivial empirical baseline; zero explanatory capability.
Baseline 2: Dosage-Only Polynomial	NS Dosage only (cubic fit)	0.715 ± 0.042	0.0384	Fails under cross-dosage validation; cannot predict the 2.0% peak or 3.0% drop when omitted.
Baseline 3: Single-Descriptor Linear	$K_{\mathrm{IC},\mathrm{ITZ}}^{\mathrm{micro}}$ only	0.812 ± 0.033	0.0315	Captures interfacial correlation, but underestimates fracture energy dissipation in the matrix.
Proposed Surrogate (XGBoost)	All 5 micro-descriptors	0.878 ± 0.024	0.0286	Optimal non-linear mapping; captures inter-phase synergistic toughening and threshold jumps.

## Data Availability

The data that support the findings of this study are available from the corresponding author upon reasonable request.

## Abbreviation

Abbreviation	Full Name/Definition
AD	Applicability Domain
ANN	Artificial Neural Network
BSE	Backscattered Electron
CH	Calcium Hydroxide
CI	Confidence Interval
C-S-H	Calcium Silicate Hydrate
FEG-SEM	Field Emission Gun Scanning Electron Microscopy
Gf	Macroscopic Fracture Energy
HRTEM	High-Resolution Transmission Electron Microscopy
Hs	Scratch Hardness
KICmacro	Macroscopic Effective Fracture Toughness
KICmicro	Microscopic Fracture Toughness derived via nanoscratch
LMM	Linear Mixed-Effects Model
LODO-CV	Leave-One-Dosage-Out Cross-Validation
MAE	Mean Absolute Error
ML	Machine Learning
MLR	Multiple Linear Regression
micro-ITZ	Clinker–Hydrate Interfacial Transition Zone at the microscale
NS	Nano-Silica
RFE	Recursive Feature Elimination
RMSE	Root Mean Squared Error
SAXS	Small-Angle X-Ray Scattering
SD	Standard Deviation
SHAP	SHapley Additive exPlanations
SVR	Support Vector Regression
UCPs	Unhydrated Cement Particles
VIF	Variance Inflation Factor
w/b	Water-to-Binder Ratio
Ws	Scratch Tangential Dissipated Work
XGBoost	eXtreme Gradient Boosting
